# The speed of internationalization in regionally clustered family firms: a deeper understanding of innovation activities and cluster affiliation

**DOI:** 10.1007/s10037-023-00182-9

**Published:** 2023-03-14

**Authors:** Telma Mendes, Vítor Braga, Carina Silva, Alexandra Braga

**Affiliations:** 1 CIICESI—Center for Research and Innovation in Business Sciences and Information Systems, School of Technology and Management (ESTG), Polytechnic Institute of Porto (P. PORTO), Porto, Portugal; 2 University of Vigo, Pontevedra, Spain

**Keywords:** Clusters, Family firms, Post-internationalization speed, Innovation activities, Cluster affiliation, D22, M16, O31, R12.

## Abstract

This article aims to explore how innovation activities and cluster affiliation moderate the relationship between family involvement and post-internationalization speed in family firms. Based on a sample of 639 Portuguese family businesses (FBs) created and internationalized between 2010 and 2018, we show that, all things being equal, higher levels of family involvement in ownership and management lead to a lower post-internationalization speed. When considering the effect of cluster affiliation, we found that clustered FBs have a lower propensity to slow down the post-internationalization process than their non-clustered counterparts. Likewise, when we account for the interactive effect of innovation activities, the results confirm that innovative FBs are less likely to slow down the post-internationalization process compared to non-innovative FBs. In addition, the concern of family firms in developing innovation was particularly pronounced when they belong to clusters. This finding is explained by the existence of socially proximate relationships with other cluster members, based on similarity, trust, knowledge exchange, and sense of belonging, which push family firms to innovate to counteract the detrimental effects of a higher family involvement in the post-internationalization speed. Our study, therefore, stresses the importance of clusters and innovation activities in moderating the relationship between family involvement and post-internationalization speed.

**Supplementary Information:**

The online version of this article (10.1007/s10037-023-00182-9) contains supplementary material, which is available to authorized users.

## Introduction

Internationalization is a key strategy for family firms to ensure long-term competitiveness and business continuity for the next generations (Arregle et al. 2012; De Massis et al. 2018). However, internationalization strategies also involve higher levels of risk and uncertainty, increasing the complexity of tasks and costs (Fernández and Nieto 2006). Hence, the number of studies focusing on the challenges faced by family firms in the context of a globalized market has notably increased (De Massis et al. 2018), transcending the family business (FB) field to the broader research domain of international business (Alayo et al. 2021; Debellis et al. 2021). The extant literature has reported that family involvement in the firm positively affects internationalization (e.g., Carr and Bateman 2009; Chen et al. 2014; Rienda et al. 2020; Zahra 2003), while other studies revealed a negative influence (e.g., D’Angelo et al. 2016; Fernández and Nieto 2006; Gómez-Mejía et al. 2010; Ray et al. 2018), and a few reported nonlinear effects (e.g., Liang et al. 2014; Sciascia et al. 2012), or no effect at all (e.g., Kano and Verbeke 2018; Pinho 2007). Nevertheless, despite the increase in academic publications, there is still a little connection between the theories of international business and family firms (Cesinger et al. 2016).

Previous research acknowledges that a family firm “*is a business governed and/or managed with the intention to shape and pursue the vision of the business held by a dominant coalition controlled by members of the same family […] in a manner that is potentially sustainable across generations of the family*” (Chua et al. 1999, p. 25). These enterprises usually internationalize following the internationalization pattern predicted in the Uppsala model (Johanson and Vahlne 1977), with exports being the most widely used entry mode (Majocchi et al. 2018). Thus, family firms usually start exporting to international markets that are geographically and culturally close (Kontinen and Ojala 2010a; Pukall and Calabrò 2014), facilitating the learning and establishment of relationships (Alayo et al. 2022). However, the Uppsala model does not differentiate between family and non-family firms in their internationalization processes (Arregle et al. 2017; Kontinen and Ojala 2010a; Pukall and Calabrò 2014). According to Arregle et al. (2017), the influence of family involvement on the decision-making process makes the family firms’ internationalization unique. While non-family firms frequently make decisions about foreign expansion based on potential financial gains and losses, family firms must balance the potential gains and losses that this strategy entails based on both financial and non-financial motives, the latter one comprising socioemotional wealth (SEW) (Gómez-Mejía et al. 2018). Thus, although the extant literature tries to analyze the consequences of the relationship between family firm governance and internationalization (e.g., Arregle et al. 2012; Cerrato and Piva 2012; Chen et al. 2014; Fernández and Nieto 2006; Sciascia et al. 2012; Zahra 2003), there is still a limited understanding on which emotional attachment factors influence family firms’ internationalization processes.

To address the aforementioned research limitations, we aim to deepen the study of family firms’ internationalization by considering the Uppsala model and the SEW perspective to explore how FBs behave after the first international market entry, focusing on the post-internationalization speed. The speed of internationalization occupies a central position as a measure of international business success (Acedo and Jones 2007; Casillas and Acedo 2013), being considered the most relevant time-based dimension in the firm’s internationalization research (Prashantham and Young 2011). According to Casillas and Acedo (2013, p. 6), the speed of internationalization is understood as *“a relationship between time and company’s international events”*, which allows to evaluate how the post-internationalization process unfolds over time. This is an extremely important issue in FBs research because, most studies measuring different dimensions of the internationalization process, are based on indicators that are reported to a certain fixed, static point of time (e.g., Chen et al. 2014; Graves and Thomas 2006; Lin 2012; Ray et al. 2018; Rienda et al. 2020; Stieg et al. 2017; Zahra 2003) informing about the level of internationalization in the family firm, rather than its post-internationalization speed. We, therefore, argue that family firms with a higher family involvement in ownership and management may be inclined to avoid internationalization if they consider that it may threaten SEW endowment and its non-financial goals. The fear of failing in foreign operations, and thus, losing their SEW with damage to the family name and organizational reputation can be disastrous (Alayo et al. 2022), thereby slowing the internationalization process described in the Uppsala model (Johanson and Vahlne 1977), which results in a more gradualist approach to international markets.

We also link our baseline proposition with the embeddedness perspective—which states that individual behaviors and choices are conditioned by social influences emerging from a flow of interactions and shifting relationships with others (Granovetter 1985)—to hypothesize that the relationship between family involvement and post-internationalization speed is contingent on the level of family firms’ embeddedness in clusters. Because of the firm-specific social capital and strong embeddedness in local networks, family firms are better positioned to leverage the spatially bounded flow of knowledge, resources, and information when belonging to clusters, resulting in a higher likelihood to internationalize (Amato et al. 2021b). Following this reasoning and adding the role of innovation activities, we suggest that this is especially true when FBs are highly innovative since the connection of family firms with other cluster members are likely to materialize (Kim et al. 2020), thereby enhancing their commitment to internationalization. This happens because innovation provides a way for family firms to explore new opportunities in the international markets (Alayo et al. 2021), and the local institutions of the cluster ecosystem play a fundamental role in their long-term development (Ricotta and Basco 2021).

Based on the above, this study explores how innovation activities and cluster affiliation moderate the relationship between family involvement and post-internationalization speed. To test our hypotheses, we relied on a sample of 639 Portuguese family firms created and internationalized between 2010 and 2018. Portugal represents a particularly suitable setting for the purpose of our study for three main reasons. First, family firms account for nearly 70% of all firms and contribute to 65% of the Portuguese gross domestic product (GDP) (AEF 2020). Second, because Portugal accounts for 19 clusters geographically dispersed in the national territory (IAPMEI 2019), where most of its small and medium-sized enterprises (SMEs) display a significant share of exports (INE 2022). Third, Portugal is a small open economy characterized by its strong innovation index (European Innovation Scoreboard 2020). Considering the innovation production in SMEs, Portugal assumes a leadership position by presenting highest shares of innovative products and business processes (Mendes et al. 2021a).

Our findings indicate that, all things being equal, higher levels of family involvement in ownership and management slow down the post-internationalization process of family firms. While cluster affiliation *per se* does not influence the post-internationalization speed, its combination with the family involvement provides a marker between two types of firms—clustered and non-clustered FBs. Indeed, clustered family firms are found to be 5.6% less likely to slow down the post-internationalization process than their non-clustered counterparts. Moreover, when developing innovation activities, innovative family firms were 6.8% less likely to decelerate the post-internationalization process when compared to non-innovative FBs. Finally, it is through cluster affiliation that the influence of innovation activities between family firms in the post-internationalization speed was particularly evident. Specifically, we found that the probability to decelerate the post-internationalization process of the innovative FBs was nearly 10 percentage points below that of non-innovative FBs when family firms belong to clusters.

This study makes theoretical and practical implications. First, it contributes to the family business literature by integrating the SEW perspective into the Uppsala model. On this basis, it shows that family involvement in ownership and management can shape the post-internationalization process. This way, our research is set with the research stream based on bringing SEW insights to internationalization theory (Alayo et al. 2022), unveiling that the family firms’ internationalization pattern fits within the Uppsala model—i.e., a higher family involvement in the family firms leads to a lower post-internationalization speed. Second, our study contributes to the convergent efforts to link regional and family business studies, trying to address the *context-less* gap (e.g., Amato et al. 2022, 2021c; Basco et al. 2021b), by introducing the role of clusters to explain the family firms’ internationalization patterns. For FBs, clusters arise not only as a socio-spatial platform but also as symbolic and emotional structures inside of which these organizations evolve across generations. Therefore, bringing the “cluster affiliation” to the study of family firms, accounts for the existence of physical, socio-institutional, and historical attributes that overlap with the attributes of the family and the firm and can, ultimately, influence the FBs internationalization pathway. Third, following the debate on the locational effect on innovation in the context of family firms (Pucci et al. 2020), we reveal the conditions under which the favorable attitudes towards innovation are likely to materialize. The study shows that cluster affiliation helps family firms to capitalize on their unique characteristics (e.g., long-term orientation) to build successful innovation which affects the post-internationalization speed.

Finally, this article has practical implications for policymakers. Our findings suggest that any public incentive that attempts to foster firms’ foreign participation and regions’ international competitiveness (Bannò et al. 2015) cannot neglect the role of family firms play (Basco and Bartkevičiūtė 2016). In this perspective, the position of family firms in clustered networks provides an advantage in intercepting and fruitfully exploiting information on internationalization practices, thus, reducing the FB’s risk perception towards internationalization. Any public intervention requires specific policies and actions that need to take into consideration the type of actors that make up the regional structure and their interaction with the geographical space. Therefore, public policies should account for the heterogeneity of economic actors in clusters (e.g., family vs. non-family firms, small vs. large firms, manufacturing vs. service firms) when tailoring policy interventions.

This paper develops as follows. First, by providing an overview of the literature linking family firms with internationalization, as well as by disentangling the effects of innovation activities and cluster affiliation on the FBs post-internationalization speed, we present the background used for hypotheses development. In the next section, we describe the sample, the measurement of the variables, and the statistical method used for data analysis. Finally, we present and discuss our results, concluding with the main contributions and suggesting some avenues for future research.

## Theoretical background and hypotheses development

### The speed of internationalization through the lens of international business research

The concept of speed of internationalization is an important issue for firms that are entering international markets (Chetty et al. 2014). Several studies have been considering “time” as the only dimension of speed measuring how long it takes to firms initiate the internationalization process (e.g., Acedo and Jones 2007; Zucchella et al. 2007). Nevertheless, this is a limited perspective because “time” might not fully capture how internationalization evolves (Aygoren and Kadakal 2018; Hilmersson and Johanson 2016). Based on this acknowledgement, Casillas and Acedo (2013) proposed a definition that embraces the relationship between time and firms’ international activities.

The depth of foreign activities and the geographical diversification across different markets are relevant sources of learning in the internationalization process (Casillas and Moreno-Menéndez 2014). Prashantham and Young (2011) considered that the *speed of country scope* (i.e., number of countries) and the* speed of international commitment* (i.e., percentage of foreign revenue) are two dimensions that reflect the firm efforts in the post-internationalization stage. Within this research stream, several expressions have been used to address the post-internationalization speed—for instance, “accelerated internationalization” (e.g., Pla-Barber and Escribá-Esteve 2006), “growth in the number of international regions” (e.g., Bloodgood 2006), and “degree of internationalization” (e.g., Cerrato and Piva 2015).

Although the speed of internationalization is the most widely used terminology (e.g., Casillas and Moreno-Menéndez 2014; Hilmersson and Johanson 2020; Vermeulen and Barkema 2002), its multidisciplinary creates conflicts in the establishment of a consensual definition (Mendes et al. 2021b; Silva et al. 2021). In addition, the current confusion is aggravated by the difficulty in explaining what the speed of internationalization should actually measure because, despite its increasing research, the extant literature has employed a wide range of measures (Hilmersson et al. 2017) considering “time to event” and “event per time” as exchangeable metrics (Johanson and Kalinic 2016). Overall, in the international business literature, we can find three dimensions covering the entire internationalization process: (1) earliness of internationalization, (2) post-internationalization pace, and (3) post-internationalization speed.

The first dimension—earliness—is usually conceptualized by the time taken between the firm’s founding and the first international market entry (e.g., Acedo and Jones 2007; Cesinger et al. 2013; Sapienza et al. 2005). The second dimension—post-internationalization pace—is understood as the time required to reach a specific degree of internationalization or performance level in international markets (Zhou 2007). The third dimension—post-internationalization speed—reflects how the depth (i.e., international scale) and the breadth (i.e., international scope) of the internationalization process, as well as the level of resource commitment abroad in terms of foreign direct investment (FDI), change over time. It is worth noting that, the earliness of internationalization and the post-internationalization pace account for the amount of time until a certain event occurs (*time to event*), while the post-internationalization speed reflects the change in the internationalization patterns denoting the relationship between international events and time (*event per time*).

These considerations are pertinent in our framework to study the family firms’ internationalization as a dynamic process, applying the multidimensional nature of the speed of internationalization (e.g., Casillas and Acedo 2013; Chetty et al. 2014; Zucchella et al. 2007). The coexistence of history dependency, plus progressive past reconstruction in the present and for the future (Vahlne and Johanson 2017), is distinctively true for family firms. A dynamic approach will, therefore, illustrate how FBs internationalization changes over time (Santangelo and Meyer 2017), by emphasizing the post-internationalization speed, which is a measure of *international events per time.*

### A brief overview on family firm internationalization

The majority of existing research assumes that the unique features of family firms influence their international scale (Arregle et al. 2021). However, there is no consensus about which of these features facilitates or constrain internationalization (Arregle et al. 2007). Several scholars have been adopting a socioemotional wealth (SEW) perspective to contend that family members prioritize the preservation of families SEW. SEW embraces “*the non-financial aspects of the firm that meet the family’s affective needs, such as identity, the ability to exercise family influence, and the perpetuation of the family dynasty*” (Gómez-Mejía et al. 2007, p. 106). Previous research (e.g., Chirico et al. 2020; Gómez-Mejía et al. 2007) presented evidence pointing that family owners and managers are so averse to the loss or reduction of SEW that they are willing to sacrifice a certain percentage of profit to preserve it. Nevertheless, the influence of this loss-aversion on the internationalization of family firms is not clear. According to Gómez-Mejía et al. (2010), SEW pulls family firms in two opposite directions, because internationalization lowers both business risk—which helps to preserve SEW—and family control—which reduces SEW. However, these authors found that, on average, family involvement is associated with a lower international scale. Other SEW related studies (e.g., Bannò and Trento 2016; Dou et al. 2019; Kraus et al. 2016; Ray et al. 2018) reached similar results, when analyzing the depth of the internationalization process towards exports.

On the other hand, unlike broader international business literature, most of the research on family firms does not conceptually or empirically distinguish international scale from scope (Arregle et al. 2021). Hence, the theoretical mechanisms, contingencies, and variables aforementioned are, for the most part, assumed to the family firms’ international scope. However, some studies, specifically account for the breadth of the internationalization process (i.e., scope), proposing more precise and robust rationales and delivering empirical results for this internationalization dimension. Based on the stewardship theory, Zahra (2003) found that the effects of family ownership and management on international scope differ. While ownership exerts a positive effect, management has a negative one. Overall, most researchers offer four theoretical arguments that can explain a lower international scope in family firms. First, Xu et al. (2020) argue that increasing the level of geographical diversification creates higher demands on resources which, in turn, rises the risk of SEW losses. Second, family owners and managers are more likely to use their networks to facilitate internationalization (Cesinger et al. 2016). However, since these networks tend to be limited and regionally bound, international scope can be lower (Tsang 2020) or constrained to a specific region (Banalieva and Eddleston 2011). Third, increased international diversity requires a higher foreign experience on the part of family leaders, as well as access to additional resources and capabilities (Arregle et al. 2021). Nevertheless, strong family social capital can hinder international scope by creating a mismatch between the competencies available in the family members’ networks and the growing diversity needed for increasing international scope, which reinforces the liabilities of foreignness for FBs (D’Angelo et al. 2016; Stadler et al. 2018). Finally, strong family social capital supports the perpetuation of the founder imprint on strategy across the next generations of leadership, which can constrain changes in the internationalization breadth (Suman 2017).

Another strand of the literature is particularly focused on exploring the level of resource commitment abroad and the timing of family firm internationalization. While it is usually assumed that family firms internationalize slowly and follow a stepwise internationalization pattern (e.g., Graves and Thomas 2008; Kontinen and Ojala 2010b, 2012), studies focusing on these dimensions suggest that the process can be more nuanced. For example, Lin (2012) found that family ownership increases the average number of foreign subsidiaries per year, but throws off its international rhythm (i.e., internationalization becomes more irregular). Similarly, Kontinen and Ojala (2012) have suggested that a higher level of family ownership in the next generations positively influences the level of resource commitment in international markets. Moreover, Stieg et al. (2017) concluded that the timing of internationalization (i.e., earliness) is linked to generational successions, but the level of resource commitment abroad is determined by the successor’s foreign experience and education level.

Based on the mixed evidence around the three dimensions reflecting the same phenomenon—i.e., the family firms’ behavior in the post-internationalization stage—we summarize core findings and identify sources of inconsistency across FBs studies in Table 1.

**Table 1:** An overview of the family firm internationalization (adapted from Arregle et al. 2021, pp. 1164–1168)

	International Scale ^a^	International Scope ^b^	Resource Commitment Abroad ^c^
Core Findings	Family firms have financial, managerial, and international knowledge constraints that restrict international scale. FBs focused on SEW display a lower international scale due to increased SEW loss that internationalization entails. Concentration of family control creates agency conflicts between majority family shareholders and minority shareholders which dampen international scale. Stewardship behavior helps overcome the challenges of internationalization. Unique resources (e.g., social capital, reputation, long-term orientation) facilitate the increase of international scale	Stewardship scholars state that family ownership positively affects international scope. Family management has a negative impact on the internationalization breadth because of the family members’ loss aversion. Increasing the level of geographical diversification leads to higher demands on resources, requires greater international experience, and rises the probability of SEW losses. Family members’ social capital is regionally bounded and lacks diversity, limiting international scope	Family firms internationalize slowly, following a gradualist approach to foreign markets. The earliness of family firm internationalization does not fall into a distinct pattern. Family ownership speeds up the level of resource commitment abroad, but negatively effects the family firm international rhythm (internationalization becomes more irregular)
Sources of Inconsistency	Diverging theoretical rationales and assumptions (e.g., stewardship theory, SEW perspective, and agency theory). The same theory (e.g., social capital) can explain positive and negative effects of family involvement in ownership and management on international scale. The use of different measures of internationalization and distinct concepts of family firms lead to conflicting and inconclusive findings. Sample may not be representative (single-country investigations yield diverging results). Insufficient consideration of family firm heterogeneity and different risk profiles of internationalization	The use of different family firm definitions. The failure in clearly distinguishing between family ownership, management, influence, and control. Lack of nuanced differentiation within central constructs (e.g., among different types of external actors, different objectives for the family firm, among others)	The internationalization patterns (including the earliness of internationalization) are context specific. Distinct methodologies and different definitions of *level of resource commitment abroad *and *rhythm.* Differences in features related to other aspects of internationalization (e.g., international scale and scope)
Conclusions	Family firms possess unique features and resources that impact international scale differently from other firms. However, extant literature fails in showing whether this uniqueness facilitates or constraints internationalization. The above contradiction is particularly evident in studies that try to establish a generalized relationship between family ownership-management and internationalization scale. The main reason for the conflicting results is due to a general lack of contextual considerations and insufficient incorporation of family firms’ heterogeneity in exploring the nature of this relationship	In the studies focusing on international scope, a greater degree of consensus exists. The majority of research states that higher levels of family involvement in the firm lead to a lower international scope	Studies that consider heterogenous contextual characteristics (at the internal and external levels), arrive at nuanced conclusions that challenge the baseline assumption that FBs internationalize slowly. However, establishing a generalized patter for temporal features of family firms’ internationalization is not possible without considering contextual differences. The diverging results at the timing of internationalization, level of resource commitment abroad, and rhythm of internationalization stem from contextual differences related to other aspects of internationalization. For instance, FBs with narrow international scope can pursue more rapid internationalization than FBs with greater international scope

^a^ International scale represents the percentage of foreign sales compared to total sales

^b^ International scope accounts for the number of countries/regions where the firm operates

^c^ The resource commitment abroad considers the level of foreign direct investment (FDI)

### Family firms and post-internationalization speed

The literature on international business has undertaken a detailed analysis of why firms engage in foreign operations, the types of resources and capabilities necessary to enter international markets, and their preferred entry modes (Alayo et al. 2022). At the same time, many theoretical perspectives have been used to analyze the internationalization of family firms (e.g., stewardship theory, SEW perspective, and agency theory) (Arregle et al. 2021). However, the Uppsala model has been mostly used to explain how FBs internationalization unfolds over time (e.g., Alayo et al. 2022; Kontinen and Ojala 2010a; Rondi et al. 2020).

According to the Uppsala model, internationalization is seen as an evolutionary process of sequential stages based on the knowledge and experiential learning of new markets (Kontinen and Ojala 2010b). Family firms usually follow this internationalization pattern, starting their foreign activities in markets that are geographically and culturally close and resorting to low resource-intensive entry modes (Calabrò and Mussolino 2013; Claver et al. 2007). Subsequently, as family firms acquire knowledge and experiential learning of foreign markets, the scope of their international activities gradually increases (Kontinen and Ojala 2010b). The patterns of global, stepwise expansion in FBs are attributed to the challenge of overcoming resource constraints and acquiring the managerial skills, knowledge, and experience needed to compete in international markets (Minetti et al. 2015). Overall, family firms internationalize in controlled ways protecting their independence, the family influence in the organization, and managing international risk, while learning from past decisions (Cesinger et al. 2016; Moreno-Menéndez and Castiglioni 2021). However, the original Uppsala model should be complemented with the SEW perspective to explain the specific behavior of family firms during the internationalization process (Alayo et al. 2022; Cesinger et al. 2016; Stieg et al. 2018), given that the Uppsala model does not consider the social, emotional, and effective endowments vested in the family firm (Berrone et al. 2012; Gómez-Mejía et al. 2018).

According to the SEW literature, family members are motivated by financial and non-financial goals, and their decision-making process depends on the reference point that dominates the final decision to be made (Gómez-Mejía et al. 2007; Zellweger et al. 2012). If family owners and managers perceive a possible threat to their socioemotional endowment, they can consider the possibility of financial losses, prioritizing socioemotional or non-financial goals over financial ones (Gómez-Mejía et al. 2007). This family-oriented particularistic behavior (Carney 2005) can lead FBs to be less internationalized (Gómez-Mejía et al. 2011), with the SEW approach explaining that this reluctance “*originates from the dominance of socio-affective utilities in family firms*” (Cesinger et al. 2016, p. 587).

This research stream acknowledges that family firms are not risk-averse organizations *per se,* but they are loss-averse when it comes to the protection of their SEW endowment (Gómez-Mejía et al. 2007). The desire to safeguard SEW explains why FBs make decisions that are not always economically justified (Arzubiaga et al. 2021; Ray et al. 2018), and also clarifies why the relevance of non-financial goals and the SEW preservation may conflict with the family firms’ internationalization process (Cesinger et al. 2016; Gómez-Mejía et al. 2011). As Zellweger et al. (2012) pointed out, once family members adopt SEW as a reference point, their focus is based on the emotional endowment that they attach to the firm. For example, maintain a good reputation built and sustained over generations can imply that the owning family prefers to avoid practices that can damage their image, such as a hypothetical failed internationalization effort (Cabrera-Suárez et al. 2014; Dyer 2021). In this situation, some FBs might be more cautious and adopt a conservative position toward internationalization for the fear of failure (Alayo et al. 2022).

Considering that the desire to safeguard family firms SEW negatively affects the resource allocation for international expansion (Scholes et al. 2016), FBs might prefer lower levels of internationalization to protect their socioemotional endowments (Gómez-Mejía et al. 2010). Thus, internationalization can be approached with more caution at a slower pace (Moreno-Menéndez and Castiglioni 2021) or even completely avoided (Stieg et al. 2018). In this case, the internationalization process proposed by the Uppsala model may suffer a slow down or setback (Alayo et al. 2022). Thus, our baseline assumption is as follows:

#### Baseline assumption

*A higher level of family involvement in ownership and management slows down the post-internationalization speed in family firms*.

However, the aforementioned assumption is mostly *context-less* (e.g., Amato et al. 2022, 2021c; Basco et al. 2021b), since it ignores the local embeddedness and business networks that can be established, for example, in clusters, which potentially influence family firms’ attitudes towards internationalization. Moreover, some firm-specific characteristics may explain the varying strength of family influence on internationalization, diminishing their loss aversion in relation to the preservation of SEW endowments. One of these firm-specific characteristics is the development of innovation in family firms (e.g., Duran et al. 2015; Xiang et al. 2019). Hence, to better understand the relationship between family involvement and post-internationalization speed, in the following subsections, we focus on innovation activities and cluster affiliation to disentangle how and when they condition the family firms’ post-entry speed.

### Innovation activities, family firms and post-internationalization speed

Innovation is essential for family firms to remain competitive and to ensure their survival in an increasingly dynamic environment (Heider et al. 2022). There are strong theoretical foundations to believe that FBs may encounter many difficulties in responding to innovation (König et al. 2013). As a traditional or even conservative organizations are unwilling to break away from existing manners of doing business in addition to their resource dependence, inertia, and rigidity, family firms are further constrained by generational transition and the pursuit of non-financial goals (Kotlar et al. 2018), which together influence how FBs manage innovation (König et al. 2013).

Although these arguments, some FBs are amongst the most innovative firms in the world (De Massis et al. 2018; Urbinati et al. 2017), because their long-term orientation acts as a stimulus to develop innovation (Diaz-Moriana et al. 2020). Some scholars have been arguing that family firms reveal lower *innovation inputs* (e.g., R&D investments) (De Massis et al. 2018; Calabrò et al. 2019), but they can produce higher *innovation outputs* (e.g., new patents or products) (De Massis et al. 2013; Urbinati et al. 2017). This happens because family members can obtain more return on their investments (Duran et al. 2015), using them to acquire differentiating technology and develop new products (Xiang et al. 2019). Thus, the desire to avoid uncertainty motivates FBs to ensure an efficient or *parsimonious* (Carney 2005) conversion of *innovation input* into *innovation output *(Duran et al. 2015; Heider et al. 2022; Matzler et al. 2015; Uhlaner 2013).

In addition to the several sources and types of innovation, there is a further differentiation in terms of innovation levels (i.e., incremental or radical) and its magnitude (i.e., exploitative or explorative) (Sharma and Salvato 2011). Because of their long-term orientation and their unique human and social capital (Sharma and Salvato 2011; Hiebl 2015), family firms are particularly well-equipped for exploiting opportunities in domains close to their existing operations through the pursuit of incremental innovations. These conditions lead family firms to innovate more incrementally^1^ rather than radically^2^ (e.g., Calabrò et al. 2019; Nieto et al. 2015; Roessl et al. 2010; Wright et al. 2016), and to perform particularly well in the domain of exploitative innovations (Bammens et al. 2015).

According to Berrone et al. (2010), family firms have the intention to preserve SEW even if such effort will make the firm miss financial opportunities. However, this SEW conservation may also have a “dark side” because it can function as a driver of self-serving behavior in a such way that some FBs place family needs above those of the firm; strong family bonds, family firm identity, and family control can encourage family members to ignore and even harm non-family stakeholders (Kellermanns et al. 2012). Moreover, family control and strong identification with the FBs can cause heirs to feel locked into and dependent upon the family and the firm, feeling suffocated and smothered by an omnipresent family and pressures to align with family decisions (Schulze et al. 2001). In this regard, FBs seldom to conduct radical innovation because it might create changes that jeopardize family interests (e.g., social identity, ownership and control, continuity of the business) (Berrone et al. 2012).

Nevertheless, Leppäaho and Ritala (2022) found that family firms pursue a wide range of responses, that may involve a change of behavior from risk-averse to risk-taking. They found that FBs are also able to develop radical innovations to tackle environmental changes. For example, to address the coronavirus-related fears, FBs invested in an intensive and proactive communication with their employees, facilitated remote work, and introduced major changes towards digitalization (Kraus et al. 2020). Accordingly, this evidence suggests that FBs managers recognize the relevance of radical innovation although, due to the desire of passing the firm to the next generation, incremental innovation is more likely to be adopted as a renewal strategy (Chrisman and Patel 2012).

Based on agency and stewardship theories, Kellermans et al. (2012) showed that higher innovativeness in FBs is associated with a superior performance. Successful dynastic families follow long-term strategies and innovate through entering new markets and applying new technologies (Bergfeld and Weber 2011). Family firms, therefore, seek for new markets, businesses, and processes, in order to guarantee the firm’s succession (Nordqvist et al. 2013). In this context, they should be able to create respectful market positions and develop creative innovations to ensure longevity and success (Ramadani et al. 2015). According to Braga et al. (2017), innovation appears in FBs as an effective business strategy, in which firms achieve greater competitive advantage, implementing new production processes, products, and/or preparing for new markets.

Donckels and Frohlich (1991) argued that innovation and internationalization arise in family firms, mostly, due to the search for business sustainability and the development of corporate processes, in order to counteract their rigidity. Moreover, Ratten and Tajeddini (2017) found that innovation serves as a mean for FBs to grow their businesses internationally, arguing that the long-term viability of a family firm demands a focus on innovation. This is due to innovation provides a way for FBs to explore new international market opportunities. In a similar vein, Alayo et al. (2021) also confirmed that family firms need to focus on exploratory and exploitative innovations to obtain competitive advantage in foreign markets and, thus, increase their internationalization level. Their study suggested that, to improve the effect of innovation on internationalization, family owners should consider involving new generations and non-family managers in the decision-making process.

In the light of the abovementioned arguments, we infer that a high level of innovation on FBs will increase the family firm willingness to internationalize, resulting in a greater ability to move quickly in international markets.

Hence, our first hypothesis is as follows:

#### *Hypothesis 1*

*Innovation activities influence the relationship between family involvement and post-internationalization speed in a such way that the post-internationalization speed is higher for innovative family firms than for non-innovative family firms*.

### Clusters, family firms and post-internationalization speed

The issue of industrial location has gained increasingly relevance after the seminal work of Alfred Marshall (1890), who recognized that the clustering of activities in a specific geographical area represents an important source of externalities (Vom Hofe and Chen 2006). According to Porter (2000, p. 16), clusters^3^ are “*geographic concentrations of interconnected companies, specialized suppliers, service providers, firms in related industries, and associated institutions (e.g., universities, standard agencies, trade associations) in a particular field that compete but also cooperate*”. The definition of clusters, therefore, builds on three key dimensions (Porter and Ketels 2009): (1) *geographic dimension, *because clusters arise due to externalities that depend on regional proximity, (2) *activity dimension*, as they encompass activities in different industries that are interconnected with each other, and (3) *business environment dimension, *since they are affected by cluster-specific conditions that are the result of actions taken by the private sector (e.g., firms), government agencies, universities, and other public institutions, acting individually and collectively. Therefore, the Porterian cluster serves to identify key issues in the competitive advantage of clustered firms. If a firm’s activities can be viewed as a number of value chain activities, then its main strategic decisions consist of placing each activity within the most adequate local environment; hence, the cluster framework is a theory of the firm which explains why some firms are more successful than others (Ortega-Colomer et al. 2016).

Based on the above, we also conclude that clusters embody a combination of competition and cooperation. Vigorous competition involves attracting new customers and retaining them. Because of the presence of multiple rivals and strong incentives, the intensity of competition among clustered firms is often accentuated (Porter, 2000). Yet, cooperation must occur in a variety of areas. Much of it is vertical—i.e., buyer-supplier—with related industries and local institutions. According to Porter (2000), competition and cooperation can coexist in clusters because they are on different dimensions, or because cooperation at some levels is part of winning the competition at other levels. This leads to the concept of coopetition defined as “*[…] a paradoxical relationship between two or more actors simultaneous involved in cooperative and competitive interactions […]*” (Bengtsson and Kock 2014, p. 182). This understanding of coopetition can be adapted to clusters—the focal firms are considered as both cooperation partners and competitors, cooperating in some activities and competing in others (Virtanen and Kock 2022). In this regard, Gnyawali and Charleton (2018) conclude that moderate levels of competition and cooperation are more likely to positively influence value creation. A “perfect” balance, therefore, exists when the partners equally contribute to value creation (i.e., cooperate) and equally appropriate the value (i.e., compete) (Bouncken et al. 2020).

In the specific case of family firms, the local embeddedness resulting from the cluster affiliation is particularly relevant due to the strong identification with the territory (Baù et al. 2019). According to Amato et al. (2021a), the local embeddedness can be seen as the nature and the depth of firm’s ties to the local, social, and economic environment. From this perspective, the local embeddedness of family and non-family firms may differ because the former is generally regarded as being inextricably linked, physically, socially, and emotionally, to the territories in which they are located (Basco 2015). As the local embeddedness increases, family firms are more likely to take advantage of localized knowledge and resources enhancing them through training and socialization processes supported by their tacit knowledge and firm-specific assets (Block and Spiegel 2013). Thus, family firms are likely to benefit from agglomeration economies (Amato et al. 2021c; Capello 2002, 2011, 2019) because they obtain both financial and non-financial utilities arising from cluster affiliation. More specifically, FBs belonging to clusters may reconcile the tradeoffs between the pursuit of financial and non-economic goals, thus, sustaining business growth at a higher rate.

Particularly, due to social, cultural, and historical connections with the *milieu *in which are located, family firms are able to leverage tacit localized knowledge and tangible resources (Bird and Wennberg 2014), as a result of several mechanisms (Amato et al. 2021b). First, families’ socially proximate relationships based on reciprocity and trustworthiness enhance interactive learning and firm’s competitive capabilities by reducing the opportunistic behavior and minimizing communication costs (Boschma 2005). Second, the co-location of family firms within a region stimulates the spontaneous development of a particular institutional setting in the form of rules and norms that regulate interactions among economic actors (Bathelt et al. 2004). Third, regionally clustered FBs are in a better position to leverage proximity dimensions (Bathelt et al. 2004; Boschma 2005; Porter 2000) which facilitate the transfer of tacit knowledge with other firms belonging to the same spatial relationships (Soleimanof et al. 2018). The strong place attachment and embeddedness in the local context allows family firms to differently exploit the advantages of co-location (Amato et al. 2021a, d; Cucculelli and Storai 2015), such as knowledge spillovers (Amato et al. 2021b), enabling economic actors to communicate, understand, and process a place-specific knowledge and information successfully (Bathelt et al. 2004).

In the international business literature, there is a growing number of evidence that cluster structures play an important role in the firm’s internationalization processes (Kowalski 2014). The analysis of clusters in fostering internationalization assumes that its resources are accessed by clustered firms improving their foreign expansion (Zen et al. 2011). Therefore, it is believed that the dynamics of clusters’ business cooperation allows the development of vital resources and collective skills for internationalization (Chetty and Wilson 2003). It follows that one firm’s action within the cluster is shaped by the attitudes of other companies in terms of information and collaborative opportunities (Amdam et al. 2020). Thus, several studies recognize that clusters act as active promoters of firm’s internationalization (Fernhaber et al. 2008; Libaers and Meyer 2011), showing that the cooperative interactions developed among clustered firms provide the resources that are needed to accelerate this process (e.g., Amdam et al. 2020; Colovic and Lamotte 2014; Felzensztein et al. 2019; Jankowska and Götz 2017; Zen et al. 2011).

For family firms, reliance on external social capital available in the cluster provides a basis for intercepting specific knowledge on international markets. In particular, by establishing new social ties or leveraging existing informal connections, FBs are able to recognize and take advantage of international opportunities (Kontinen and Ojala 2010b). For this purpose, family members can mobilize their personal contacts in both foreign and domestic contexts (Baù et al. 2019). Exploiting a dense network of relationships appears to be particularly beneficial for FBs belonging to clusters (Cucculelli and Storai 2015), where externalities arise as a driver of early and faster internationalization (Yi and Wang 2012). Because of their firm-specific social capital and strong embeddedness in local networks (Amato et al. 2021b), clustered family firms are better positioned to leverage the spatially bounded flow of knowledge and information, resulting in a higher propensity to accelerate the internationalization process.

Given the aforementioned arguments, since internationalization entails significant investments and uncertain returns to FBs, the cluster members, with a greater understanding of the international markets, will have a positive influence in shaping family members’ risk perception towards foreign expansion, reducing their unwillingness to increase the level of international commitment. Thereby, FBs belonging to clusters, when interacting with other clustered associates, improve their knowledge about foreign markets reducing the fear of the unknown. Accordingly, our second hypothesis is as follows:

#### *Hypothesis 2*

*Location in a cluster influences the relationship between family involvement and post-internationalization speed in a such way that the post-internationalization speed is higher for clustered family firms than for non-clustered family firms*.

### Innovation activities, clusters, and post-internationalization speed in family firms

Over the last two decades, clusters have emerged as a central issue in the firm’s innovation (Kowalski 2014). According to Piore and Sabel (1984), continuous innovation is an intrinsic characteristic of these structures and an essential condition for their growth. A cluster provides a set of knowledge inputs that support innovative capacity; these inputs can come from firms in related industries, suppliers, customers, competitors, universities, and public funded institutions (Feldman 1994). Previous studies also contend that face-to-face contacts and geographical proximity facilitate the diffusion of innovations (Jaffe et al. 1993). Indeed, some scholars (e.g., Baptista and Swann 1998; Bell 2005; Kowalski 2014) highlight that clusters can strengthen the firm’s innovative performance.

In investigating additional sources of knowledge and the mechanisms of learning relevant for innovation, the literature shows that the concentration of family firms in spatially bounded areas (e.g., clusters, industrial districts, or regions) provides opportunities for the transmission of knowledge (Amato et al. 2021d). Defined as free charge-knowledge flow occurring either spontaneously (i.e., without any intent) or intentionally (Kesidou and Romijn 2008), knowledge spillovers are at the foundation of agglomeration economies, allowing to achieve competitive advantages reflected in cost-saving, productivity gains, or higher innovation performance, resulting from firm’s co-location within a place or region (Galliano et al. 2015).

However, the space understood only in terms of physical distance offers a partial explanation of the mechanisms behind the dissemination of geographical-bounded knowledge flow and its influence on innovative behavior (Boschma 2005). In this perspective, the relational capital arises as a missing piece of the puzzle on firms, knowledge spillovers, and innovation (Capello 2002). In the spatial-relational approach *à la *Capello (2002), the social and relational proximity complement the classical geographical proximity underlying the diffusion of territorial knowledge relevant for FBs innovation. Known as “local buzz” (Bathelt et al. 2004), the social dimension of proximity refers to the network of communication and information linkages arising from face-to-face contacts, co-presence, and co-location of firms within the same place or region, which promotes the exchange of knowledge and new ideas relevant for innovation (Kesidou and Romijn 2008).

According to Arregle et al. (2007), the long-term, reciprocal, and trustworthy relationships among family members tend to be replicated outside the organizational boundaries, shaping in a unique way how family firms interact with their local setting (Backman and Palmberg 2015). The economic activity of FBs is strongly embedded in a stable and durable set of social relations (Baù et al. 2019) that provides access to critical tangible and intangible resources (Backman and Palmberg 2015). Specifically, the centrality of family members in their social and business networks is found to facilitate the access and the exchange of external valuable resources—such as, business opportunities (Zahra 2010) and up-date information (Salvato and Melin 2008)—relevant for innovation (Calabrò et al. 2019).

Family firm owners, as “dedicated owners” (Porter 1992), will pursue the development of lasting innovation cooperative relationships with other local firms and organizations. The FBs long-term orientation makes them attractive partners for such cooperation. For instance, in times of financial stress, family firms are considered reliable cooperation partners who are less likely to cut investments in research and end promising networks; moreover, because of their local roots and strong ties with local partners, they are also less likely than other firms to act opportunistically (Block and Spiegel 2013). According to Block (2010), family owners have solid local networks and have built strong ties with the environment in which they are headquartered, because this territory represents the place in which they grew up. The long-term orientation, local roots, and strong regional embeddedness of family firms supports them in identifying valuable sources of knowledge and strengthening the regional innovation system, which leads to higher levels of innovation output in FBs (Cooke 2001).

Previous studies exploring how family firms behave in clusters found that both regional density and industry positively affect regional entrepreneurship (Cappelli et al. 2021), with the FBs internationalization being strongly impacted by local ties (Ranfagni et al. 2021). This, therefore, implies that local institutions play a fundamental role on the adoption and development of growth strategies in family firms (Ricotta and Basco 2021). To summarize, we argue that the FBs ability to think in the long-term and the linkages established in clusters favor cooperation for developing innovation and creating knowledge spillovers, thereby, strengthening regional internationalization systems. In other words, the high level of innovation promoted by the cluster atmosphere functions as a driver of FBs internationalization. Based on these arguments, we can infer that the moderating effect of innovation activities on the relationship between family involvement and post-internationalization speed is further strengthened when family firms belong to clusters. Hence, our third hypothesis is as follows:

#### *Hypothesis 3*

*The moderating effect of innovation activities on the relationship between family involvement and post-internationalization speed is stronger in clustered family firms than in non-clustered family firms*.

**Fig. 1:**
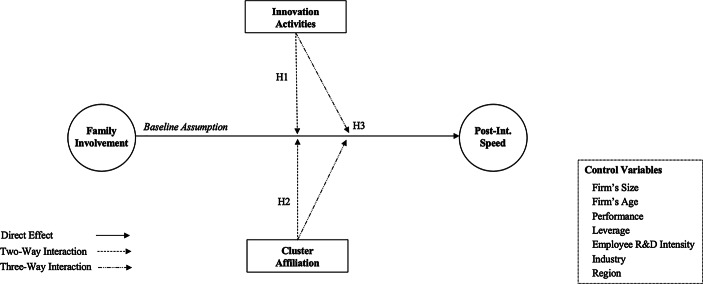
Research model. *Note*: On the one hand, family involvement and post-internationalization speed are the constructs or latent variables (i.e., variables that are not directly measured) represented by circles. These constructs have a measurement model that specifies the relationship between each construct and its indicator variables (i.e., family involvement is measured by family ownership and family management, while post-internationalization speed is measured by the change in international scope and scale). On the other hand, innovation activities and cluster affiliation are observed variables represented by rectangles

Figure 1 summarizes the proposed relationships.

## Methodology

### Data collection and sample

**Fig. 2:**
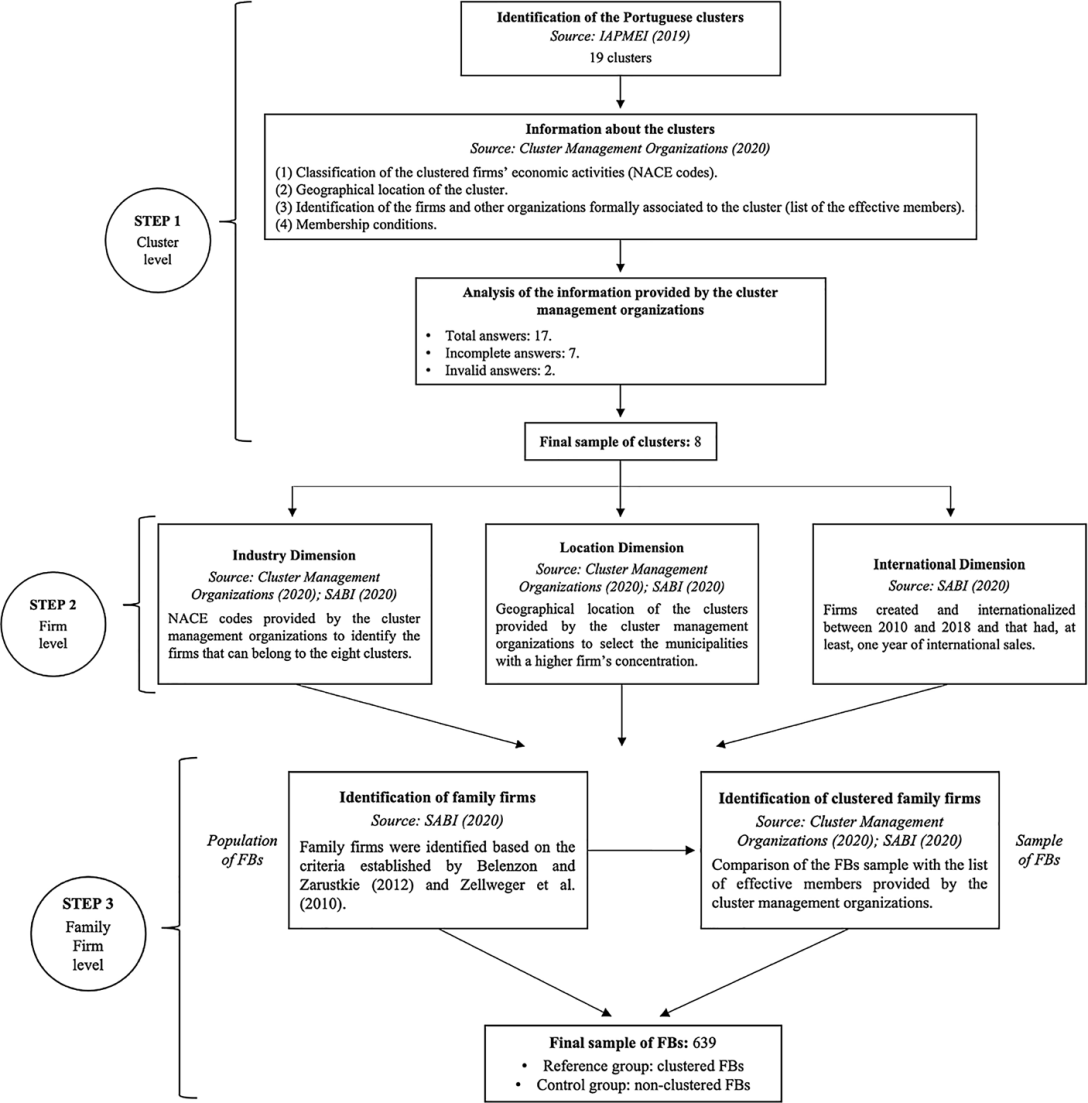
Design of the study data collection

The data collection process involved three different steps (Fig. 2). The first step consisted of identifying the Portuguese clusters because, to select the firms that may belong to them, we needed to have access to the NACE codes required by each of the cluster management organizations. To this purpose, we consulted the IAPMEI website (Agency for Competitiveness and Innovation) which allowed us to identify 19 clusters at the national level. Then, we contacted all the cluster management organizations to request the following information: (1) classification of the clustered firms’ economic activities (NACE codes), (2) geographical location of the cluster, (3) identification of the firms and other organizations (e.g., universities, research centers, public authorities, among others) formally^4^ associated to the cluster, and (4) membership conditions. The initial contact was made via email and, later, by telephone, to reinforce the request for participation in the study conducted between October 2019 and February 2020. A total of 17 answers from the cluster management organizations was received, of which 9 were excluded because 7 were not complete and 2 did not match with the firms’ NACE^5^ codes available on the secondary database selected to retrieve quantitative data—SABI database^6^. Thus, eight^7^ clusters remained for analysis: (1) Footwear and Fashion, (2) Textile—Technology and Fashion, (3) Automotive, (4) Engineering & Tooling, (5) PRODUTECH Production Technologies, (6) Vine and Wine, (7) Petrochemical, Industrial Chemistry and Refining, and (8) Smart Cities Portugal.

In the second step, we used the SABI database to collect quantitative data for the firms that can belong to the eight clusters listed above. Drawing on the clustering literature (e.g., Baptista and Swann 1998; Fernhaber et al. 2008; Porter and Ketels 2009), we established three different criteria to select those firms. First, we adopted the NACE codes provided by the cluster managing organizations (Baptista and Swann 1998; Fernhaber et al. 2008; Porter and Ketels 2009) to obtain the firms that match with the industry sectors required by each of them (i.e., industry dimension). These firms are manufacturers and service providers stemming from different sectors—footwear, textile, winery, chemical, molds, plastic, automotive, and production technologies. Second, based on the clusters’ geographic dimension (Porter and Ketels 2009), we used the information provided by the cluster management organizations about the geographic location of the clusters, to select the municipalities with a higher firm’s concentration at the regional level (i.e., location dimension). Third, since we are interested in studying the post-internationalization speed, the selected enterprises were created and internationalized^8^ between January 2010 and December 2018 and had, at least, one year of international sales (i.e., international dimension).

The last step involved the identification of the family firms through the information available on SABI database. Family firms were classified according to the criteria proposed by Belenzon and Zarustkie (2012) and Zellweger et al. (2010), which are explained in the following subsection. At the date^9^ of data extraction (May 2020), 639 FBs met both criteria. Then, we have confronted our sample of family firms with the list of effective members provided by the cluster management organizations, which showed the firms that were formally associated to the eight clusters under analysis. Through this matching, we were able to obtain our reference group (clustered FBs) and the control group (non-clustered FBs). Table 2 shows the geographical location of family firms organized by NUTS^10^, districts, and municipalities, letting to visualize the regions in which they are concentrated.

**Table 2:** Sample representativeness by NUTS, districts, and municipalities

NUTS II	NUTS III	Districts	Municipalities	Family Firms Sample (*n* = 639)	
				n	%
North	Metropolitan Area of Porto (17 municipalities; 1,721,038 habitants; 2041.3 km^2^)	Porto	Porto	136	21.3
			Vila Nova de Gaia	36	5.6
			Maia	1	0.2
		Aveiro	Santa Maria da Feira	18	2.8
			Oliveira de Azeméis	3	0.5
	Tâmega e Sousa (11 municipalities; 418,018 habitants; 1831.5 km^2^)	Porto	Felgueiras	34	5.3
	Ave (8 municipalities; 413,262 habitants; 1451.4 km^2^)	Braga	Guimarães	46	7.2
	Cávado (6 municipalities; 403,922 habitants; 1245.8 km^2^)	Braga	Braga	29	4.5
			Barcelos	26	4.1
	**North (4 NUTS)**			**329**	**51.5**
Metropolitan Area of Lisboa	Metropolitan Area of Lisboa (18 municipalities; 2,840,006 habitants; 3015.2 km^2^)	Lisboa	Lisboa	251	39.3
			Oeiras	43	6.7
			Sintra	2	0.3
		Setúbal	Almada	4	2.2
	**Metropolitan Area of Lisboa (1 NUTS)**			**310**	**48.5**
**Total**				**639**	**100.0**

*Source*: Based on Pordata (2020)

### Variables

#### Dependent variable

Post-internationalization speed is a multidimensional metric^11^ (Casillas and Acedo 2013; Chetty et al. 2014) measured by the changes registered in international scale and scope. Following previous studies (e.g., Banalieva et al. 2022), we measured the change in* international scale *with the following formula: $\frac{(\frac{\text{Foreign}\,\text{Sales}_{t}}{\text{Total}\,\text{Sales}_{t}})-(\frac{\text{Foreign}\,\text{Sales}_{t-1}}{\text{Total}\,\text{Sales}_{t-1}})}{(\frac{\text{Foreign}\,\text{Sales}_{t-1}}{\text{Total}\,\text{Sales}_{t-1}})}$. However, based on the limitations of the foreign sales to total sales ratio (FSTS) as a measure of international scale (Verbeke and Forootan 2012), to exclude exceptional periods in the family firms’ international activities, this formula was applied between eight consecutive years (2010–2018) since, some of the sampled firms, did not reveal foreign sales in all years under consideration (e.g., several were created during those years). We, therefore, obtained the average growth rate by summing all the rates divided by an eight-year period. On the other hand, the change in* international scope *reflects the geographical diversification of FBs foreign activities (Cerrato and Piva 2012; George et al. 2005), proxied by whether the family firm only sells to the European Union (EU), non-EU, or both geographical markets. The EU market includes the 28 state-members^12^, while non-EU markets account for other European countries and the remaining worldwide regions (America, Asia, Africa, and Oceania). This variable was coded^13^ with values ranging from 1 to 3 (1 = no diversification vs. 3 = highly diversified).

#### Independent variable

Definitions of family firms differ widely across the literature (Arregle et al. 2017; Hennart et al. 2019; Schulze et al. 2001). While studies contend that family ownership is the defining proxy to consider a firm as family business (e.g., Carr and Bateman 2009), others suggest that family firms must display substantial levels of family ownership and management to satisfy the criterion (Alayo et al. 2019). Other scholars differentiate family-controlled firms (firms controlled by families due to the high level of ownership) from family-influenced firms (firms in which family owners and managers display less control) (Sirmon et al. 2008). Hence, the debate to define FBs is still open (Arregle et al. 2021). Although different studies have been trying to clarify the definition by developing measurement scales, the heterogeneity of family firms makes it difficult to reach a consensus (Pearson et al. 2014). Given that SABI database does not distinguishes between family and non-family firms, this study follows the criteria^14^ proposed by Belenzon and Zarustkie (2012) and Zellweger et al. (2010). Their approach established two requirements for defining a business as a family firm:

1. First, we excluded all firms with a single shareholder considering as FBs the organizations where, at least, two shareholders have the same name and hold 50% or more of the equity (Belenzon and Zarutskie 2012). The SABI offers the possibility of automatic data searches using the option “shareholder one or more known individuals or families”. Based on this criterion, another one is added “global ultimate owners” allowing to obtain the final shareholder or owner of each firm. This criterion (“global ultimate owners”) can be crossed with the percentage of ownership by combining the indicators of independence that SABI provides, allowing to select the firms where 50% or more of the ownership belongs to one family, physical person, or legal entity. Finally, the same surname among shareholders, chief executive officers (CEO), and directors board members, involves requesting from SABI—legal form, global parent shareholder, immediate shareholder, CEO, and directors.
2. Second, to increase the FBs number, we also included the organizations whose corporate name contained the reference “… and Sons”, “… and Brothers”, “… and Heirs”, “… and Successors”. This criterion relates to the organizational identity where it is common to find, in the firm’s corporate designation, the family name or the reference to family ties (Zellweger et al. 2010).

We, thus, consider as family firms a business in which most of the equity (i.e., ≥ 50%) is owned by, at least, two individuals in the family sharing the same last name. Accordingly, family involvement is a reflective latent variable operationalized using two items: (1) *family ownership *indicating the percentage of equity hold by family members (e.g., Chen et al. 2014; Sciascia et al. 2012), and (2) *family management *using a dummy variable, which assumes the value of 1 when family members occupy executive positions and 0 otherwise (Ray et al. 2018).

#### Moderating variables

Considering the aim of this study, to identify the entities that may belong to clusters we adopted the NACE codes (Baptista and Swann 1998; Fernhaber et al. 2008) provided by their managing associations, including a dummy variable (1 = if the family firm belongs to the cluster; 0 = otherwise) to classify the FBs that were formally associated to these structures^15^ (Bell 2005; Zucchella et al. 2007). With regards to innovation activities, they were measured as the innovation output (e.g., De Massis et al. 2013; Urbinati et al. 2017) using the number of registered brands. The extant literature acknowledges that patent counts, as the most popular measure for firm innovation (Wan et al. 2005), are directly intertwined to inventiveness (Walker 1995). Due to severe data limitations in terms of innovation output, the number of patents was proxied by the registered brands hold by family firms. Innovation output is, therefore, a measure of innovation activities, regardless of being radical (development of new brands) or incremental (slight improvements in existing brands). For this research, a dummy variable was created taking the value of 1 if the family firm has, at least, one registered brand and 0 otherwise.

#### Control variables

To deal with unobserved heterogeneity, we controlled for a wide set of variables potentially affecting the post-internationalization speed. Previous research highlights that larger FBs display greater financial and non-financial resources that promote internationalization (Chen et al. 2014). To control for this effect, we measured the *firm’s size* through the number of employees (Hilmersson 2014). Similarly, older FBs display a higher ability to collect information about international operations building infrastructures that allow a successful internationalization (Ray et al. 2018). On this basis, *firm’s age *is controlled and measured by the number of years that the family firm has been in operation (Kowalik et al. 2017). Considering that firm’s *performance* assumes a key role in the decision-making process, it was also included as a control variable represented by the return on assets (ROA) (Lin 2012). As post-internationalization speed is usually influenced by the firm’s financial distress, we introduced *leverage *measured as the firm’s book value of total debt to total assets (Lins et al. 2013). To account for the firm’s human capital, we controlled *employee R&D intensity *(Baù et al. 2019) operationalized by the average number of full-time employees developing R&D activities. Finally, we also included dummy variables to control for the *industry *(e.g., Mendes et al. 2021b) and *region *(Amato et al. 2021c) effects. To summarize, Table 9 provides complete information about the measurement of the variables.

### Statistical analysis

To test the proposed hypotheses, we used the partial least squares structural equation modeling^16^ (PLS-SEM) that allows to estimate complex interactions between observed and latent variables. The few FBs studies that include latent variables have been specifying composites of multi-item scales (typically sum scores) as inputs for regression analyses (i.e., sum scores regression) (Basco et al. 2021a). While common, this practice is problematic because it ignores the attenuating effect of measurement error inherent in this approach. Several studies have shown that the failure to correct measurement errors can produce a combination of under- or over-estimation effects regarding the relationships between constructs (e.g., Hair et al. 2017; Yuan et al. 2020). Conversely, PLS-SEM allows measurement errors to be reduced (Henseler et al. 2014). That is, rather than considering all aspects covered by the indicator weights as equally important, as in sum scores regression, PLS-SEM weights the indicators individually, depending on their explanatory power in downstream model relationships (e.g., Jöreskog and Wold 1982). PLS-SEM also runs partial regressions, but the parameter estimation follows an iterative process accounting for the entire model structure (Sarstedt et al. 2020a).

In addition, PLS-SEM based endogeneity assessment allows FBs researchers to correct biases in the model estimation caused by omitted variables (Hult et al. 2018). Updated guidelines for PLS-SEM models evaluation consider recent developments in validity assessment (e.g., Hair et al. 2019, 2020; Sarstedt et al. 2020a), including approaches dealing with unobserved heterogeneity (e.g., Hair et al. 2016; Matthews 2017). These extensions not only facilitate a more holistic assessment of research results—for example, in terms of the model’s predictive power—but also enable scholars to consider new research contexts in their PLS-SEM analysis. According to Hair et al. (2021), researchers in the area of FBs, particularly those trying to advance this field applying the methods that best fit their research and objectives, should consider these extensions.

- This method works efficiently with secondary data and when used to estimate path models comprising many indicators, constructs, and relationships (Hair et al. 2019; Sarstedt et al. 2014).
- PLS-SEM supports both explanatory and predictive goals when analyzing the model’s causal-predictive relationships (Chin et al. 2020).
- This type of estimation is especially suited to the development of new theories, as well as the extension of existing ones (Richter et al. 2016).
- PLS-SEM has a satisfactory functioning with large (Hair et al. 2019) and small sample sizes (Sarstedt et al. 2014).
- This technique allows to account and estimate the effects of moderator variables (Becker et al. 2012; Henseler and Chin 2010).
- PLS-SEM allows to correct the data when the variables included in the analysis do not follow a normal distribution^17^ (Hair et al. 2019; Nitzl 2016).

Thus, the SmartPLS 3.3.9 software^18^ was used to estimate our model (Ringle et al. 2015). While the sign and significance of the coefficient of the variable *family involvement *is related to the baseline assumption, hypothesis, 1, 2, and 3 are operationalized by the following interaction terms: family involvement * innovation activities, family involvement * cluster affiliation, family involvement * innovation activities * cluster affiliation. For the sake of clarity, we interpret the interaction terms by group comparisons (Matthews 2017). Given the existence of as many groups as possible combinations, a specific reference group was identified^19^. The sign and statistically significance of the marginal effect of a given group in comparison with the reference group provides straightforward evidence of differences across groups (Amato et al. 2021c). Therefore, to investigate the relationship between family involvement and post-internationalization speed contingent to innovation activities, cluster affiliation, and both instances, the groups family involvement * non-innovative family firms, family involvement * non-clustered family firms, family involvement * non-innovative family firms * non-clustered family firms were compared with the reference groups family involvement * innovative family firms, family involvement * clustered family firms, family involvement * innovative family firms * clustered family firms, respectively.

For a greater specification in the determination of sample size, we calculated the statistical power. The analysis allows to determine the sample size required to develop the study. According to Cohen (1992), the value of the statistical power should be 0.80 or higher, with a significance level of 5%. Based on the effect size value (f^2^ = 0.15) and the number of predictors, the statistical power for the full sample was estimated using G * Power 3.1.9 software^20^ (Faul et al. 2014). We chose the F‑test analysis selecting the post-hoc option for “linear multiple regression: fixed model, R^2^ deviation from zero”. Using these settings, the statistical power is greater than 0.80 for all groups—full sample (*n* = 639), innovative FBs (*n* = 106), non-innovative FBs (*n* = 533), clustered FBs (*n* = 98), and non-clustered FBs (*n* = 541)—confirming that significant relationships can be identified on the data, and the sample size is sufficient for the magnitude of the effects found (1 − β > 0.80, α error prob = 0.05, and effect size = 0.15). Additionally, computing the type of “a priori” power analysis (1 − β > 0.80, α error prob = 0.05, and effect size = 0.15), with the independent (family involvement) and moderator (cluster affiliation and innovation activities) variables in our model, it resulted in a required total sample size of 77 firms, so even the smallest subsample (clustered FBs = 98) exceeds the minimum sample size.

## Results

### Descriptive statistics

Table 3 provides means, standard deviations, and Pearson correlation coefficients. This statistical analysis was conducted in the IBM SPSS statistics 28 software^21^. As outlined in this table, the correlations between variables are relatively low, suggesting that multicollinearity does not affect our results. Regarding common method bias (CMB), which is a potential problem when the predictor and criterion variables are obtained from the same data source (Basco 2013), we used two procedures to control and detect CMB. First, we ran a factor analysis (Harman’s single factor test) by introducing all variables (i.e., dependent, independent, moderating, and control variables) (Podsakoff et al. 2003). A method factor did not emerge; thus, we conclude that CMB was not a real problem in this study. Second, following Kock (2015), we conducted a test based on collinearity assessment. This procedure aims to analyze if the variation inflation factors (VIF) are above 3.3, indicating pathological collinearity in the data. We analyzed VIF values in the partial regressions and found that they were clearly below to the cutoff value of 3.3 (Table 7). Hence, this result is consistent to the one produced by the Harman’s single factor test, suggesting that common method bias was not a real concern.

**Table 3:** Descriptive statistics and Pearson correlations

	Mean	S.D.	Min	Max	1	2	3	4	5	6	7	8	9	10	11
1. Cluster Affiliation	0.150	0.361	0	1	1	–	–	–	–	–	–	–	–	–	–
2. International Scope	1.920	0.874	1	3	0.054	1	–	–	–	–	–	–	–	–	–
3. Innovation Activities	0.163	0.392	0	1	0.041	0.181^*^	1	–	–	–	–	–	–	–	–
4. International Scale	0.446	4.292	−0.496	94.309	0.047	0.069	0.237^*^	1	–	–	–	–	–	–	–
5. Firm’s Size	4.860	10.597	1	121	0.109^*^	0.141^*^	0.049	0.064	1	–	–	–	–	–	–
6. Performance	8.443	137.103	−1599.48	2511.44	−0.022	−0.082^*^	−0.022	−0.012	−0.015	1	–	–	–	–	–
7. Family Ownership	96.213	10.370	50.97	100.00	0.021	−0.076^+^	−0.019	−0.089^+^	−0.110^*^	0.056	1	–	–	–	–
8. Family Management	0.150	0.359	0	1	0.026	−0.073^+^	0.056	−0.079^+^	0.016	−0.004	−0.067	1	–	–	–
9. Firm’s Age	4.320	2.463	0	8	0.131*	0.170^*^	0.184^*^	0.106^*^	0.227^*^	−0.030	−0.040	0.063	1	–	–
10. Employee R&D Intensity	7.432	15.558	0	62	−0.024	0.036^+^	0.085^+^	0.064^+^	−0.030	0.012	0.005	0.012	0.052^+^	1	–
11. Leverage	26.639	5.622	0	89.239	0.002	0.009	0.052	0.006	0.042	0.023	0.001	0.013	0.008	0.019	1

Mean, standard deviation (S.D.), minimum (min), and maximum (max) values. *p*-values significant at ^+^ *p* < 0.05, ^*^ *p* < 0.01

In our sample, while family firms are relatively widespread in terms of geographical overseas activity—the average firm is slightly diversified and sells for non-EU markets—the level of sales is relatively balanced between domestic and international markets (average FSTS ratio = 45%). On average, the family firms are profitable in terms of the usage of assets—ROA (8.4%). The average FBs have roughly 4 years old, and they are mostly small by employing around 5 employees. Finally, the family firms have, on average, 7 employees developing R&D activities, with the total debt accounting for almost 27% of total assets.

### Measurement checks

Exploratory factor analysis was conducted to assess the reliability and validity of the latent variables using IBM SPSS statistics 28 software. The results of the exploratory factor analysis are presented in Table 4. The measure of adequacy of the Kaiser-Meyer-Ohlin (KMO) compares simple correlations with partial correlations. Our output resulted in a KMO of 0.518 meeting the KMO criteria between 0.5 and 1 (Kaiser 1958). Furthermore, the Bartlett’s sphericity test verifies that the correlation matrix is an identity matrix which would imply that its intercorrelations are zero. This test takes a value of 350,339 (6 d.f.) with a *p*-value below to the significance level of 0.001. This means that the observed variables are correlated justifying the use of factor analysis. On the other hand, the diagonal of the anti-image matrix contains the measures of sample adequacy (MSA), comparing the magnitude of the coefficients of the observed correlations with the magnitude of the coefficients of the partial correlations, in which all variables must reveal MSA values above 0.50 (Hair et al. 1999). Since none of the observed variables had MSA values below to 0.5, it was not necessary to remove any of them. The communalities extracted, representing the amount of total variance of the original variables explained by the common factors (i.e., high communalities indicate the amount of variance that was extracted by the factors), returned values above 0.50 for most variables (Hair et al. 1999). Only the observed variable—change in* international scale*—showed less common variability with the others (less than 0.50) however, it was maintained in the analysis, because its MSA was above 0.50 (Table 4). The total variance explained also met the criteria of being higher than 0.5 (Hair et al. 1999).

**Table 4:** Exploratory factor analysis

Latent Variables	Observed Variables	MSA (Anti-image matrix)	Communalities extracted	Total variance explained (Principal component) (%)	Component matrix	KMO and Bartlett’s test
Family Involvement	Family Management	0.512	0.642	33.180	0.770	KMO = 0.518 Bartlett’s Test: Approx. Chi-Square = 350.339 d.f. (degrees of freedom) = 6 Sig ≤ 0.001
	Family Ownership	0.512	0.641		0.770	
Post-Internationalization Speed	International Scale	0.604	0.429	58.538	0.575	
	International Scope	0.625	0.630		0.767	

After the extraction^22^, two factors have emerged corresponding to the reflective latent variables:

- Factor 1—Family Involvement: constituted by the observed variables *family ownership *(percentage of equity hold by family members) and *family management *(dummy variable).
- Factor 2—Post-Internationalization Speed: composed by the observed variables change in* international scale *(FSTS ratio) and change in* international scope *(geographical diversification of foreign activities).

Upon the identification of which observed variables constitute the latent variables through the exploratory factor analysis, the following step was carried out in the SmartPLS 3.3.9 software adopting a rule that retained observed variables must met the minimum threshold of 0.60 (Hair et al. 2013). Since this confirmatory factor analysis is related to the evaluation of the reflective measurement models, a detailed explanation of this step can be found in the following subsection.

### Reflective measurement model assessment

The evaluation of the PLS-SEM results begins with the assessment of the reflective measurement models^23^ (i.e., family involvement and post-internationalization speed). Table 5 shows the results and evaluation criteria outcomes. In the case of reflectively measured constructs, we should start by examining the indicator loadings (outer loadings). Loadings above 0.60 indicate a sufficient level of reliability (Hair et al. 2013). Since all outer loadings range between 0.680 and 0.821, they exceed the recommended threshold. Next, we analyze the convergent validity of the latent variables. According to Sarstedt et al. (2014), convergent validity measures the extent to which a construct converges in its indicators by explaining the items’ variance. The convergent validity is assessed by the average variance extracted (AVE) for all indicators associated with a construct. An acceptable AVE is 0.50 or higher, since indicates that, on average, the construct explains over 50% of the variance of its items (Sarstedt et al. 2014). The AVE for family involvement is 0.527 and for post-internationalization speed corresponds to 0.568, revealing convergent validity (Fornell and Larcker 1981).

**Table 5:** Assessment of the reflective measurement models

Constructs	Indicators	Convergent Validity			Internal Consistency Reliability		
		Outer Loadings	Indicator Reliability	AVE	CR* ρ*_*c*_	*ρ*_*A*_	CA
Family Involvement	Family Management	0.727	0.529	0.527	0.700	0.103	0.103
	Family Ownership	0.725	0.526				
Post-Internationalization Speed	Internationalization Scale	0.680	0.462	0.568	0.722	0.252	0.243
	Internationalization Scope	0.821	0.674				

*AVE* average extracted variance, *CR* composite reliability, *CA* Cronbach’s alpha

The variables cluster affiliation, innovation activities, firm’s size, performance, firm’s age, employee R&D intensity, and leverage are not included in the analysis because they are single items

The next step involves the assessment of the constructs’ internal consistency reliability. When using PLS-SEM, internal consistency reliability is typically evaluated using Jöreskog’s (1971) composite reliability *ρ*_*c*_ (CR), where higher values indicate greater levels of reliability. According to Hair et al. (2019), values between 0.70 and 0.90 are considered satisfactory to good. All CR values (ranging from 0.700 to 0.722) were higher than the suggested threshold of 0.70. The Cronbach’s alpha (CA) is another measure of internal consistency reliability that assumes similar thresholds, but produces lower levels than CR (Hair et al. 2019). Specifically, CA is a less precise measure of reliability as the items are unweighted. Conversely, in CR the indicators are weighted based on its individual loadings and, thus, the items’ reliability is higher than in CA (Hair et al. 2019). The CA values suggest that the constructs family involvement and post-internationalization speed are inadmissible measures (in line with Hair et al. 2019). While CA may be too conservative, the CR can be too liberal, and the construct’s true reliability is typically viewed as within these two extreme values. As an alternative, Dijkstra and Henseler (2015) proposed *ρ*_*A*_ as an approximately exact measure of construct reliability, which usually lies between CA and CR. In our case, *ρ*_*A*_ are also below to the recommended cutoff value of 0.707 (Dijkstra and Henseler 2015). However, considering the explanatory nature of this research, the lower values of CA and *ρ*_*A*__,_ and the acceptable levels of AVE and CR *ρ*_*c*_, allow to proceed with the analysis (Hair et al. 2010).

Once the reliability and convergent validity of the reflective constructs are successfully established, the next step involves assessing the discriminant validity (Table 6). According to Sarstedt et al. (2014), discriminant validity determines the extent to which a construct is empirically distinct from other constructs in the path model. The most conservative technique to evaluate discriminant validity is the Fornell and Larcker (1981) criterion. This method compares each AVE values with the squared inter-construct correlation (a measure of shared variance) of that latent variable with all other constructs in the structural model. In our sample, the correlations between the pair of constructs did not exceed the square root of AVE (Fornell and Larcker 1981).

**Table 6:** Assessment of discriminant validity

**Fornell and Larcker** (1981) **criterion**			**HTMT ratio** (**Henseler et al.** 2015)	
	1	2		Family Involvement
1. Family Involvement	*0.726*	–	Post-Internationalization Speed	0.432 [0.247; 0.529]
2. Post-Internationalization Speed	−0.055	*0.754*		

The italic numbers on the diagonal are the square root of AVE. Off-diagonal values is the correlation between the latent variables (family involvement and post-internationalization speed). The values in the brackets represent the 95% confidence intervals. The variables cluster affiliation, innovation activities, firm’s size, performance, firm’s age, employee R&D intensity, and leverage are not included in the analysis because they are single items

Nevertheless, recent research indicates that, this metric, is not suitable for discriminant validity assessment. For instance, Henseler et al. (2015) showed that the Fornell and Larcker criterion does not perform well, particularly, when the indicator loadings vary slightly (i.e., when they range between 0.65 and 0.85). Based on this limitation, Henseler et al. (2015) proposed the hetero-trait mono-trait (HTMT) of the correlations. The HTMT is defined as the mean value of the item correlations across constructs relative to the (geometric) mean of the average correlations for the items measuring the same latent variable (Hair et al. 2019). For variables that are conceptually distinct, Henseler et al. (2015) recommended a conservative threshold of 0.85 for the HTMT correlations between latent variables. Additionally, bootstrapping can also be applied to test whether the HTMT value is significantly different to 1 (Henseler et al. 2015). In our analysis, we conclude that the HTMT correlation for the relationship between post-internationalization speed and family involvement is below to the cutoff value of 0.85. We also ran the bootstrapping procedure with 5000 samples choosing the bias-corrected and accelerated (BCa) bootstrap and the one-tailed testing at 5% significance level. The results reveal that the HTMT value is significantly different from 1, which means that discriminant validity has been established between the pair of constructs. The reflective measurement models, therefore, indicated that the measures displayed satisfactory levels of reliability and validity, allowing to proceed to the structural model evaluation.

### Structural model assessment

The second step of the PLS-SEM analysis involves the assessment of the structural model. Unlike covariance-based structural equation modeling (CB-SEM), PLS-SEM does not provide a standard goodness-of-fit statistic^24^, and efforts for establishing a corresponding one have proven to be highly problematic (Henseler and Sarstedt 2013). Instead, the assessment of the model’s quality is based on its ability to predict the dependent constructs. The assessment of the structural model involves evaluating: (1) the relevance and significance of path coefficients (β), (2) the in-sample explanatory power (R^2^ e f^2^), and (3) the out-of-sample predictive power (Q^2^). Moreover, prior to this evaluation, the structural model must be assessed for potential collinearity in the partial regressions (Sarstedt et al. 2014).

The estimation of the path coefficients relies on a series of regression analyses. Therefore, it is extremely important to ascertain whether that regression results are not biased by collinearity issues. Since all VIF values were below to the recommended threshold of 5 (Hair et al. 2022; Table 7), we conclude that multicollinearity was not a problem. Then, the strength and significance of the path coefficients was examined through the bootstrapping as the basis for calculating t‑values (Sarstedt et al. 2014). We report the results of the path coefficients analysis in Table 7. In Model 1, we introduced the family involvement construct along with control variables. The coefficient of family involvement is negative and statistically significant at the 1% level, suggesting that—all things being equal—higher levels of family involvement in ownership and management slow down the post-internationalization process of family firms (β = −0.055; *p* < 0.01), which supported our baseline assumption. By looking at the control variables, both firm’s size (β = 0.154; *p* < 0.001) and age (β = 0.114; *p* < 0.005) are positively related to the likelihood of increase the FBs post-internationalization speed. Likewise, a higher number of employees involved in R&D activities (*employee R&D intensity*) leads to an acceleration of the post-internationalization process (β = 0.043; *p* < 0.01). Conversely, neither a greater profitability (*performance*) nor the higher levels of leverage have a significant impact on post-internationalization speed.

**Table 7:** Assessment of the structural models

	Model 1			Model 2			Model 3			Model 4			Model 5		
	β	f^2^	VIF	β	f^2^	VIF	β	f^2^	VIF	β	f^2^	VIF	β	f^2^	VIF
**Firm’s Size**	0.154 (3.893^***^)	0.025	1.069	0.151 (3.889^***^)	0.023	1.069	0.149 (3.888^***^)	0.022	1.069	0.149 (3.888^***^)	0.022	1.069	0.152 (3.889^***^)	0.023	1.069
**Firm’s Age**	0.116 (3.125^**^)	0.014	1.110	0.112 (3.119^**^)	0.012	1.110	0.110 (3.117^**^)	0.011	1.110	0.110 (3.117^**^)	0.011	1.110	0.113 (3.120^**^)	0.012	1.110
**Performance**	0.023 (0.512)	0.001	1.001	0.020 (0.499)	0.001	1.001	0.019 (0.497)	0.001	1.001	0.019 (0.497)	0.001	1.001	0.022 (0.510)	0.001	1.001
**Leverage**	0.012 (0.229)	0.000	1.005	0.011 (0.227)	0.000	1.001	0.010 (0.226)	0.000	1.001	0.010 (0.226)	0.000	1.001	0.011 (0.227)	0.000	1.001
**Employee R&D Intensity**	0.043 (2.439^*^)	0.002	1.021	0.041 (2.435^*^)	0.002	1.021	0.040 (2.434^*^)	0.002	1.021	0.040 (2.434^*^)	0.002	1.021	0.042 (2.437^*^)	0.002	1.021
**Family Involvement (*Baseline Assumption)***	−0.055 (2.348^*^)	0.003	1.026	−0.055 (2.348^*^)	0.003	1.026	–	–	–	–	–	–	–	–	–
**Innovation Activities**	–	–	–	0.188 (3.942^***^)	0.038	1.041	–	–	–	0.188 (3.942^***^)	0.038	1.041	–	–	–
**Cluster Affiliation**	–	–	–	0.012 (0.270)	0.000	1.036	0.012 (0.270)	0.000	1.036	–	–	–	–	–	–
Family Involvement * Innovative Family Firms as a reference group	–	–	–	–	–	–	–	–	–	–	–	–	–	–	–
Family Involvement * Non-Innovative Family Firms *(H1)*	–	–	–	–	–	–	0.068 (2.136^+^)	0.005	1.015	–	–	–	–	–	–
Family Involvement * Clustered Family Firms as a reference group	–	–	–	–	–	–	–	–	–	–	–	–	–	–	–
Family Involvement * Non-Clustered Family Firms *(H2)*	–	–	–	–	–	–	–	–	–	0.056 (1.912^+^)	0.003	1.017	–	–	–
Family Involvement * Innovative Family Firms * Clustered Family Firms as a reference group	–	–	–	–	–	–	–	–	–	–	–	–	–	–	–
Family Involvement * Non-Innovative Family Firms * Clustered Family Firms	–	–	–	–	–	–	–	–	–	–	–	–	0.018 (0.498)	0.000	1.008
Family Involvement * Innovative Family Firms * Non-Clustered Family Firms	–	–	–	–	–	–	–	–	–	–	–	–	0.032 (1.111)	0.001	1.012
Family Involvement * Non-Innovative Family Firms * Non-Clustered Family Firms *(H3)*	–	–	–	–	–	–	–	–	–	–	–	–	0.103 (2.432^*^)	0.007	1.022
Region	Included			Included			Included			Included			Included		
Industry	Included			Included			Included			Included			Included		
R^2^	0.103			0.117			0.115			0.127			0.116		

The dependent variable is the post-internationalization speed. *VIF* inner VIF values for the partial least regressions, *R*^*2*^ explained variance of post-internationalization speed, *f*^*2*^ effect size. Path coefficients significant at *p*-values: + *p* < 0.050; * *p* < 0.010; ** *p* < 0.005; *** *p* < 0.001. The values in the brackets represent t‑values. t‑values thresholds at one-tailed test of alpha = 0.05 and 5000 resamples: t (0.05; 4999) = 1.645; t (0.01; 4999) = 2.327; t (0.005; 4999) = 2.576; t (0.001; 4999) = 3.091

In Model 2 we added the remaining two independent variables that will constitute the interaction terms with family involvement. The coefficient of cluster affiliation is positive but not statistically significant. Therefore, there is no evidence that the post-internationalization is directly affected by the degree to which family firms are anchored in clusters. In turn, the coefficient of innovation activities is positive and statistically significant at 0.1% level (β = 0.188; *p* < 0.001). Specifically, the probability to accelerate the post-internationalization process when family firms innovate is roughly 18% higher than when they do not.

In Model 3 we tested hypothesis 1 by comparing innovative family firms and non-innovative family firms at equal levels of family involvement. The marginal effect of the two-way interaction family involvement * non-innovative family firms—as opposed to innovative counterparts as a reference group—is positive and statistically significant at 5% level (β = 0.068; *p* < 0.05). This result suggests that, when it comes to post-internationalization speed, innovation activities affect family firms in different ways. In innovative family firms the probability to decelerate the post-internationalization process is 6.8% lower than their family counterparts with equal levels of family involvement and non-innovative. Thus, hypothesis 1 was supported.

In Model 4 we tested hypothesis 2 by comparing clustered family firms and non-clustered firms with the same levels of family involvement. The marginal effect of the two-way interaction family involvement * non-clustered family firms—as opposed to the clustered family firms as a reference group—is positive and statistically significant at 5% level (β = 0.056; *p* < 0.05), providing evidence that clustered and non-clustered FBs act differently in the post-internationalization process at equal levels of family involvement in ownership and management. Particularly, clustered family firms were found 5.6% less likely to slow down the post-internationalization process than their non-clustered counterparts. This evidence, therefore, gave support to hypothesis 2.

Finally, in Model 5 we tested hypothesis 3 by computing the marginal effect of the three-way interaction family involvement * non-innovative family firms * non-clustered family firms as opposed to the innovative and clustered family firms reference group. The marginal effect is positive and statistically significant at 1% level (beta value [β] = 0.103; *p* < 0.01), providing evidence that in clustered family firms, the probability to slow down the post-internationalization process is lower when FBs innovate in comparison to those that do not innovate. In particular, the likelihood of decelerate the post-internationalization process in innovative FBs belonging to clusters is 10 percentage points below that of non-innovative FBs non-affiliated to the cluster. This finding confirmed that the effect of innovation activities in the family firms’ post-internationalization speed is especially strengthened when FBs formally belong to clusters, thus, supporting hypothesis 3.

The next step involved reviewing the in-sample explanatory power (R^2^ e f^2^). The R^2^ is a measure of the variance explained in the dependent variable accounting for the model’s predictive accuracy. Our R^2^ values range between 10.3% and 12.7% (Table 7), exceeding the acceptable cutoff point of 10% (Falk and Miller 1992). Moreover, the effect size (f^2^) complements the R^2^ assessment, considering the relative impact of an independent variable on the dependent variable through the changes in R^2^ values (Cohen 1988). According to Cohen (1988), the f^2^ effect size can be classified as follows: f^2^ ≥ 0.35 (high), 0.15 ≤ f^2^ < 0.35 (medium), 0.02 ≤ f^2^ < 0.15 (small), and f^2^ < 0.02 (negligible). Overall, our f^2^ effect sizes are mostly classified as small or negligible (Table 7).

The final step requires the assessment of the out-of-sample predictive power (Q^2^). The Q^2^ builds on the blindfolding procedure, which omits a part of the data matrix, therefore, estimating the model parameters and predicting the omitted part by using the previously computed estimates (Sarstedt et al. 2014). The smaller the difference between predicted and original values the greater the Q^2^ and, hence, the model’s predictive accuracy. This analysis focused on the dependent construct and its indicators. We determined the predictive relevance by carrying out the blindfolding procedure using an omission distance of seven (D = 7; Sarstedt et al. 2014). Table 8 shows that the indicators of post-internationalization speed achieved Q^2^ values larger than zero, indicating that the model outperforms the naïve benchmark (i.e., the training sample means) (Sarstedt et al. 2021).

**Table 8:** Results of PLS_predict_

Indicators	Q^2^ Predict	RMSE	
		PLS-SEM	LM
International Scale	0.042	0.848	0.851
International Scope	0.068	0.291	0.292

*Q*^*2*^* predict* cross-validated redundancy, *RMSE* root-mean-square error, *PLS-SEM* PLS path models, *LM* linear models benchmark

To classify the model’s predictive power, we ran the PLS_predict_ with ten folds and ten repetitions (Shmueli et al. 2019). Analyzing the prediction errors produced by the PLS path models, we concluded that the distribution was not highly unsymmetric. Hence, the following analysis focused on root-mean-square error (RMSE) statistics (Table 8). The analysis showed that the RMSE values produced by the PLS-SEM is consistently lower than the one of the linear models (LM) benchmark. This evidence, therefore, suggests that the models revealed a high out-of-sample predictive power (Shmueli et al. 2019).

### Robustness check

To check the validity of the findings, further analysis^25^ was conducted. The extant literature has proposed several techniques for assessing the robustness of PLS-SEM results. These methods address both measurement and structural models (Hair et al. 2019). In terms of measurement models, Gudergan et al. (2008) have proposed the confirmatory tetrad analysis (CTA-PLS), which enables empirically substantiating the specification of measurement models (formative or reflective). The CTA-PLS relies on the concept of tetrads that describe the difference between the product of one pair of covariances and the product of another pair of covariances (Bollen and Ting 2000). However, it is worth noting that, that CTA-PLS is an empirical test of measurement models and, the primary method to determine the reflective or formative model specification, should be grounded on the theoretical reasoning (Hair et al. 2022). According to a‑priori assumption established through the literature, the latent variables—family involvement and post-internationalization speed—have reflective measurement models^26^.

In terms of structural models, Sarstedt et al. (2020b) suggest the assessment of nonlinear effects, endogeneity, and unobserved heterogeneity. First, to test for potential nonlinearities, we used the Ramsey’s (1969) test applied to the latent variables scores extracted after the convergence of the PLS-SEM algorithm. According to Hair et al. (2019), a significant test in any of the partial regressions indicates a potential nonlinear effect. The results revealed that the partial regression of the independent variables on post-internationalization speed is not subject to nonlinearities (F (2, 628) = 0.184; *p* = 0.896). We, therefore, conclude that the liner effects model was robust.

Second, when the research perspective is primarily explanatory, it is important testing the results for endogeneity (Hair et al. 2019). Endogeneity typically occurs when researchers have omitted a construct that correlates with one or more independent variables and the dependent construct in a partial regression of the PLS model (Hair et al. 2019). Our assessment of potential endogeneity follows Hult et al.’s (2018) approach, starting with application of Park and Gupta’s (2012) Gaussian copula, using the latent variables scores of the original models. The first step consists of verifying whether the variables are non-normally distributed resorting to the Kolmogorov-Smirnov test (Sarstedt and Mooi 2019). The results showed that none of the variables have normally distributed scores, allowing to proceed with Park and Gupta’s (2012) procedure. This analysis allowed us to conclude that none of the Gaussian copulas was statistically significant (i.e., the *p*-values were higher than the significance level of 5%). Considering the independent variables as potentially endogenous they revealed non-significant copulas of 0.013 for family involvement (*p*-value = 0.816), 0.461 for cluster affiliation (*p*-value = 0.167), −0.607 for innovation activities (*p*-value = 0.216), -0.127 for the interaction term family involvement * innovation activities (*p*-value = 0.120), −0.079 for the interaction term family involvement * cluster affiliation (*p*-value = 0.629), −0.010 for the interaction term family involvement * innovation activities * cluster affiliation (*p*-value = 0.751), −0.072 for firm’s age (*p*-value = 0.161), 0.054 for firm’s size (*p*-value = 0.329), 0.010 for performance (*p*-value = 0.811), −0.042 for leverage (*p*-value = 0.816), and 0.027 for employee R&D intensity (*p*-value = 0.302). We also have considered all other combinations of Gaussian copulas, and none was statistically significant. We, thus, conclude that endogeneity was not a problem in our data (Hult et al. 2018).

Finally, unobserved heterogeneity should be assessed to ascertain whether the analysis of the entire dataset is reasonable or not (Hair et al. 2019). Following Sarstedt et al. (2017b), to identify potential unobserved heterogeneity, we conducted the Finite-Mixture (FIMIX) segmentation^27^. We ran the procedure by assuming a one-segment solution, using the default settings for the stop criterion (1.0E-10), the maximum number of interactions (5000), and the number of repetitions (10) (Matthews et al. 2016). In order to determine the maximum number of segments^28^ to extract, we relied on the statistical power analysis described in subsection 3.3, suggesting a minimum sample size of 77 cases, which allowed to extract nine segments (639/77 ≅ 8.299). Hence, we executed the FIMIX-PLS for two to nine segments using the same initial default settings. The results of the fit indices suggested an ambiguously picture. According to Sarstedt et al. (2011), when the modified Akaike’s information criteria with factor 3 (AIC_3_) and the consistent Akaike’s information criteria (CAIC) indicate the same number of segments, the findings probably point to an appropriate outcome. Nevertheless, in our dataset, AIC_3_ and CAIC did not indicate the same number of segments (AIC_3_ = 7, CAIC = 3). Moreover, Hair et al. (2016) highlighted that, while AIC overestimates the correct number of segments, the minimum description length with factor 5 (MDL_5_) underestimates them. AIC_3_ suggested a seven-segment solution which means that the correct number of segments was clearly below than this. In turn, CAIC and, particularly, MDL_5_ both showed a three-segment solution, indicating that three or more segments should be considered. To address this issue, Sarstedt et al. (2011) pointed that, the modified Akaike’s information criteria with factor 4 (AIC_4_) and the Bayesian information criteria (BIC) usually perform well when are used to assess the correct number of segments. In our analysis, AIC_4_ and BIC pointed to a three segment-solution, which appeared to be densely clustered according to the entropy statistic (EN = 0.871 > 0.50) (Hair et al. 2016). Together, the results do not unambiguously underline a specific segmentation solution since AIC_3_ and CAIC pointed to a different number of segments, and MDL_5_ suggested the same number of segments as AIC_4_ and BIC. This evidence, therefore, suggests that the unobserved heterogeneity was not at a critical level on the entire dataset (Sarstedt et al. 2020b).

## Conclusions

### Discussion

Drawing on the convergence between internationalization, regional and family business studies, this study investigates the post-internationalization speed of family firms by considering the moderating effect of innovation activities and cluster affiliation. In this paper, we assume that FBs have family-oriented non-financial objectives that influence the family firm behavior. Based on previous studies, we propose that family firms are loss-averse organizations in relation to their SEW; thus, they may prefer lower levels of internationalization to protect their socioemotional endowment (Gómez-Mejía et al. 2010). When family members strongly identify with the firm, they usually develop a special concern for their reputation (Loehde et al. 2020) and, hence, the identification with the firm might not be an advantage in internationalization because these operations usually entail higher risk than operating in the home-country, increasing the probability of failure. Failing in foreign strategies not only generates financial losses but also damages the image and reputation of family firms (Pongelli et al. 2019). Under the primacy of SEW, we observe that the willingness to protect and preserve the family legacy, image, and reputation leads to a gradual involvement with international markets as proposed by the Uppsala model (Johanson and Vahlne 1977). Our findings revealed that a higher family involvement in ownership and management results in a lower post-internationalization speed. These outcomes not only validate our baseline assumption but also conform to many earlier studies showing that family firms are less likely to internationalize when compared to non-family counterparts (e.g., Arregle et al. 2017; D’Angelo et al. 2016; Graves and Thomas 2006; Hennart et al. 2019; Lin 2012).

On the other hand, our results highlight the importance of innovation activities for a greater understanding of the differences between innovative and non-innovative family firms’ post-internationalization process. Regarding the general stance towards innovation activities, family firms develop a *parsimonious *(Carney 2005) conversion of* innovation input *(e.g., R&D expenditures) into *innovation output* (e.g., patents or brands) (Duran et al. 2015; Heider et al. 2022; Matzler et al. 2015; Uhlaner 2013), which allows to enter in new markets (Bergfeld and Weber 2011). The analysis of the direct effect of innovation activities on post-internationalization speed suggests that innovation serves as a mean for family firms to grow their businesses internationally (Braga et al. 2017; Ratten and Tajeddini 2017). When we analyze innovation activities interacting with the level of family involvement in ownership and management, the results revealed different responses, with innovative family firms found to be less likely to slow down the post-internationalization process than non-innovative counterparts. This finding is consistent with previous studies highlighting that family members prioritize short-term investment to maintain current SEW endowment and receive a quick return from such investments (e.g., short-term sales growth) (Kammerlander and Ganter 2015; Sharma and Salvato 2011). In this case, the development of innovation is prioritized in daily operations (Kraiczy et al. 2014; Sharma and Salvato 2011) to assist family firms in achieving a higher level of international sales, as well as to ensure their long-term development and survival (Le Mens et al. 2015).

Moreover, the findings also pointed out the relevance of clusters to understand the differences between clustered and non-clustered family firms’ post-internationalization process. The role of clusters—understood as the geographical concentration of interconnected companies and other spatial actors that compete but also cooperate (Porter 2000)—on FBs internationalization choices have been largely overlooked. As firms “*do not exist in a vacuum devoid of connection to actual locations*” (Guthey et al. 2014, p. 259), clusters may provide a source of opportunities that help family firms to internationalize. From this perspective, internationalization choices may be contingent on the set of economic, social, and emotional connections that firms have established with their geographical and social *milieu* (Capello 2019). Although positive, the direct effect of cluster affiliation on post-internationalization speed, is not statistically significant. This insignificant effect implies that family firms at home are not necessarily faster than scattered peers in the rate of going international. Such finding is somehow consistent with previous international business studies showing that clusters by their own do not influence the speed of internationalization (e.g., Luo et al. 2005; Varma et al. 2016).

However, when we compare clustered and non-clustered family firms—with cluster affiliation interacting with the level of family involvement in ownership and management—our outcomes highlighted the importance of clusters for a greater understanding of the differences between clustered and non-clustered family firms’ post-internationalization speed. Our findings revealed that clustered FBs are less likely to slow down the post-internationalization process than non-clustered counterparts. Through the cluster affiliation, family firms are in a better position to align financial and non-financial objectives, thereby boosting a proactive behavior. Specifically, the other cluster members can shape strategy formulation and significantly contribute through their advice, experience, social capital, and knowledge, potentially improving the decision-making process in family firms, especially for risky and complex strategies such as internationalization (Zahra 2003). While clusters *per se *do not affect post-internationalization speed, in the case of family firms with a higher family involvement in ownership and management it was found to play an important role. Particularly, the strong economic links and territorial identity of family firms in clusters turn into a spatial loyalty which further mitigates the propensity to decelerate post-internationalization process when compared to non-clustered peers. Hence, as local roots provide locational advantages to family firms (Backman and Palmberg 2015; Baù et al. 2019), our findings support earlier studies showing the association between the embeddedness of family firms in the local *milieu *and their proactiveness (Berrone et al. 2010; Dekker and Hasso 2016).

Nevertheless, considering separately the effects of innovation activities and cluster affiliation offers a partial view of how organizational (i.e., innovation) and contextual (i.e., clusters) dimensions influence FBs internationalization choices. Indeed, when innovation activities and cluster affiliation are considered simultaneously^29^, we found that the innovative behavior of family firms stands out when they belong to clusters. Socially proximate relationships with the firms’ immediate surroundings, based on similarity and affective bonds push family firms to be innovative and, thus, in clustered FBs the probability to slow down the post-internationalization process is lower when they focus on innovation. The difference in the propensity to slow down the post-internationalization process between innovative and non-innovative family firms equals nearly 10% showing that innovative FBs in clusters are less likely to exhibit slower internationalization patterns. This result reveals that the amplified spatial bonds, network relationships and knowledge spillovers of family firms in clusters are extremely important to the development of innovation, which does not appear to the same extent when innovation activities and cluster affiliation are considered separately (6.8% and 5.6%, respectively). This novel finding shows the role of innovation activities in safeguarding the family firm during the internationalization process when they are formally belonging to clusters. Hence, our study confirms the uniqueness of innovative clustered family firms in limiting the detrimental effects of a higher involvement in ownership and management on post-internationalization speed.

### Contributions and policy implications

Our study has several theoretical and practical implications. From a theoretical point of view, we integrate the SEW perspective into the Uppsala model to advance our understanding of the family firms’ internationalization. In doing so, we analyze the post-internationalization speed rather than exclusively focusing on the level of internationalization in FBs. Our study, therefore, takes a step forward when compared to existing research (e.g., Chen et al. 2014; Graves and Thomas 2006; Rienda et al. 2020; Ray et al. 2018; Zahra 2003) because it incorporates the temporal dimensions of the post-internationalization speed into the FBs research, concluding that higher levels of family involvement in ownership and management slow down the post-internationalization process, which is somehow consistent with the gradual internationalization pattern proposed by the Uppsala model.

This study also contributes to the literature by explaining the causes of heterogeneity (i.e., innovation activities and cluster affiliation) among family firms in relation to their internationalization process. First, we contribute to the convergent efforts between regional and family business studies. While family business research has traditionally overlooked the regional context in which the economic activity of the firm and the social life of the family takes place, the interaction between family firms and territory is steadily emerging as the missing piece for understanding the FBs distinctiveness (Basco 2015; Stough et al. 2015). Thus, we attempt to address the *context-less *gap in FBs studies (e.g., Amato et al. 2022, 2021c; Basco et al. 2021b), by introducing the role of clusters. For family firms, clusters arise not only as a socio-spatial platform to which they are functionally and economically bounded, but also as symbolic and emotional structures inside of which these organizations evolve across generations. Therefore, introducing the “cluster affiliation” in the study of family firms accounts for the existence of physical, socio-institutional, and historical attributes that overlap with the attributes of the family and the firm and can, ultimately, influence the FBs internationalization pathway. In addition, for regional studies, the recognition of family firms enables investigations in the role of clusters as independent production factors and generator of distinctive static and dynamic advantages for family firms belonging to them. Second, following the debate into the locational effect on innovation in the context of family firms (Pucci et al. 2020), we reveal the conditions under which the favorable attitudes towards innovation are likely to materialize. While previous studies considered the characteristics of the territory where the family firm is located (Kim et al. 2020), we show that belonging to clusters helps FBs to capitalize their unique characteristics (e.g., long-term orientation, social capital) to build successful innovation which affects the post-internationalization speed.

The findings of this study also have important implications to practitioners. The SEW of family firms and their non-financial goals play a pivotal role in making strategic decisions. In some situations, financial and non-financial goals may conflict, and thus, it is important to align both objectives inside the family firm. Thus, family members should work in favor of the business, requiring a collaborative environment and a constructive debate, as well as the development of initiatives to strengthen their social capital to facilitate the exploration of international opportunities. In addition, as CEOs in family firms are key actors with an enduring presence in the business, they must collaborate with other actors outside the firm (e.g., cluster members), to reduce the detrimental effect of a higher family involvement in ownership and management on post-internationalization speed. The cluster members support and complement the profound knowledge and experience of the family owners and managers, thereby improving their strategic roles. This is important because the simple inclusion of family members does not guarantee successful results; the CEOs and family board members should be motivated and involved in the FBs activities to contribute effectively. Having motivated and identified family members on the board, developing innovation activities, and belonging to clusters helps to align business objectives with family goals and can increase the motivation of family firms to internationalize.

Finally, this article has practical implications for policymakers. Our findings suggest that any public incentive that attempts to foster firms’ foreign participation and regions’ international competitiveness (Bannò et al. 2015) cannot neglect the role of family firms play (Basco and Bartkevičiūtė 2016). In this perspective, the position of family firms in clustered networks provides an advantage in intercepting and fruitfully exploiting information on internationalization practices, thus, reducing the FBs risk perception towards internationalization. In fact, given the importance of family firms in absolute (i.e., the total number of operating businesses), and relative (i.e., the contribution to the GDP and economic well-being) terms, the proper endowment of productive factors at both regional and local levels appears to be crucial for their competitiveness and survival. In addition to this, regional governments should promote the establishment of solid collaborative linkages in an attempt to induce higher level of innovation in family firms. This happens because besides efforts internal to the firm—mainly in the form of human and financial resources devoted to innovation-related activities—innovation also depends on “*structural, institutional and relational factors that are localized and specific to geographical contexts*” (Cantner et al. 2010, p. 1939). In summary, any public intervention requires specific policies and actions that need to take into consideration the type of actors that make up the regional structure and their interaction with the geographical space. Policies with one-size-fits-all philosophy that try to boost regional internationalization, innovation, and productivity, have some limitations because not all firms act in the same way due to different objectives influencing their behavior. Accordingly, there is a need for more awareness to the unique and valuable role of FBs when policymakers design and try to implement policies to foster regional and local growth (Basco and Bartkevičiūtė 2016). In other words, the policies developed to support family firms on internationalization, similar to general entrepreneurship policies, need to be contextualized, taking into consideration the place-specific role of FBs in regional development.

### Limitations and avenues for future research

Our research has some limitations that future studies are called to address. First, we considered only one country (Portugal). Although the results can be generalized to a limited extent to other small, open, and relatively well-developed economies, future studies should expand the analysis to other countries to account for the influence of distinctive institutional and cultural settings. Second, the SABI database did not contain information about the dynamics of international scope over time and does not report other classifications than EU and non-EU markets. Thus, the post-internationalization speed of family firms is captured by the change in international scale between 2010 and 2018 (i.e., time-variant indicator), but limited by the change in international scope reported to 2018 (i.e., time-invariant indicator). A more fine-grained operationalization for international scope is warranted for future studies to analyze how family firms behave in the post-internationalization process. Similarly related to constraints on data collected, one of the main difficulties in FBs literature, relates to the use of different methods to identify family firms (Arregle et al. 2017; Hennart et al. 2019). To overcome this problem, we employed the criteria of Belenzon and Zarutskie (2012) and Zellweger et al. (2010). Nevertheless, these criteria might lead to overestimation problems, as the shareholders last names can be common without any family ties (blood or married). At the same time, when there are married bonds, but the surnames are not the same, FBs may be considered as a non-FBs with an underestimate problem. Such limitations may have led to some errors in the identification of family firms. We recognize these constraints at the conceptual level since our study entirely relies on a demographic approach^30^ to define family firms. Future research should test the consistency of our results with multiple definitions of family firms, integrating components of involvement and essence approaches accounting for *soft* factors such as the vision and intentions of family members (Basco 2013) and using, for example, the F‑PEC scale (Astrachan et al. 2002) to capture in a broader way the role of family involvement on the post-internationalization speed. Third, alternative measures of cluster affiliation can be used to investigate whether family firms’ post-internationalization speed is sensitive to other operationalizations of clusters. Specifically, alternative and complementary measures such as the location quotient (Baù et al. 2019)—largely recognized in the agglomeration literature to characterize industrial specialization (Galliano et al. 2015)—can be used in future research efforts. In addition to this, as our study develops from a micro-level approach, future studies may step into a meso-level perspective to explore the role of collective aggregate actions (i.e., family firms’ density) as a source of regional resilience (Block and Spiegel 2013), and the effects employee productivity on FBs post-internationalization speed (Bernard and Jensen 2004). Finally, although we have used the SEW perspective to conceptualize the family firms’ behavior during internationalization, this concept was not measured *per se. *To provide deeper understanding on how FBs manage the tradeoffs between financial and non-financial goals, future studies could develop case studies or in-depth interviews to complement our empirical evidence. The family firm-cluster “nexus” and the SEW perspective applied to internationalization stand out as a promising opportunity for investigation with qualitative methods, that have been proven extremely useful in developing new theories and testing existing ones.

## Supplementary Information

Robustness Check

## Appendix

**Table 9:** Description of variables

Variables		Measurement	Theoretical Foundation	Proxy	Source
Dependent Variable	Post-Internationalization Speed	Change in international scale	e.g., Banalieva et al. (2022), Gómez-Mejía et al. (2010), Rienda et al. (2020)	(FSTS_t_ − FSTS_t‑1_) / FSTS_t‑1_	SABI (2020)
		Change in international scope	e.g., George et al. (2005), Cerrato and Piva (2012)	Firms were coded: 1 = no diversification (only selling to the EU market), 2 = slightly diversified (only selling to non-EU markets), 3 = highly diversified (selling for both markets)	SABI (2020)
Independent Variable	Family Involvement ^a^	Family ownership	e.g., Chen et al. (2014), Sciascia et al. (2012)	Percentage of equity hold by family members	SABI (2020)
		Family management	e.g., Ray et al. (2018)	Dummy variable: 1 = when family members occupy executive positions, 0 = otherwise	SABI (2020)
Moderating Variables	Cluster Affiliation	Firms that may belong to the cluster	e.g., Baptista and Swann (1998), Fernhaber et al. (2008)	NACE codes	IAPMEI (2019) SABI (2020)
		Firms formally associated to the cluster	e.g., Bell (2005), Zucchella et al. (2007)	Dummy variable: 1 = when family firms belong to the cluster, 0 = otherwise	SABI (2020)
	Innovation Activities	Innovation output	e.g., De Massis et al. (2013), Urbinati et al. (2017)	Dummy variable: 1 = when family firms hold, at least, one registered brand, 0 = otherwise	SABI (2020)
Control Variables	Firm’s Size	According to the EU recommendation 2003/361	e.g., Chen et al. (2014), Hilmersson (2014)	Number of employees	SABI (2020)
	Firm’s Age	Time since firm’s foundation until the latest year available on the database (2018)	e.g., Kowalik et al. (2017), Ray et al. (2018)	Number of years	SABI (2020)
	Performance	Return on assets (ROA)	e.g., Lin (2012)	Ratio between net income and total assets	SABI (2020)
	Leverage	Firm’s book value	e.g., Lins et al. (2013)	Ratio between total debt and total assets	SABI (2020)
	Employee R&D Intensity	Firm’s human capital	e.g., Baù et al. (2019)	Average number of full-time employees developing R&D activities	SABI (2020)
	Industry	NACE codes	e.g., Mendes et al. (2021b)	Dummy variables	SABI (2020)
	Region	Geographical location	e.g., Amato et al. (2021c)	Dummy variables	SABI (2020) Pordata (2020)

^a^ *Family-owned firms:* all the firms are family-owned since we considered as FBs the firms in which most of the equity (i.e., ≥ 50%) was hold by family members. However, family firms display varying levels of family ownership: 50–60% (*n* = 12), 61–70% (*n* = 39), 71–80% (*n* = 16), 81–90% (*n* = 16), 91–100% (*n* = 556)

*Family-managed firms: *the distribution of family members in management also varies according to the level of family ownership: 50–60% (*n* = 2), 61–70% (*n* = 11), 71–80% (*n* = 2), 81–90% (*n* = 3), 91–100% (*n* = 79). A higher concentration of family managers occurs in FBs with higher levels of family ownership (i.e., family ownership >90%)

*Family-owned and managed: *within our sample of family firms, 15.2% (*n* = 97) of FBs are owned and managed by family members. In this case, the average of shareholding owned by the family is roughly 96.21%

## Footnotes

1 Incremental innovation consists mainly of improvements and line extensions through fitting, recombining, reusing, and adapting current knowledge (Colombo et al. 2017). This type of innovation offers a modest novelty to existing products and services in the current markets (Slater et al. 2014).

2 Radical innovation is defined as the commercialization of an entirely novel idea, which is new to the markets (Colombo et al. 2017). This type of innovation offers potentially higher customer benefits over previous products and services in the industry (Kyriakopoulos et al. 2016).

3 This definition is compatible with the theoretical perspective of clusters as structures aggregating the geographical proximity (e.g., Saxenian 1994) and the interorganizational networks (e.g., Rosenfeld 2005). Accordingly, clusters are understood to occur on the actor level (clustered firms’ interactions), while also being embedded in specific geographical areas (Porter 1998).

4 According to the managing associations, to be considered a cluster member the firms must fulfill the following criteria: (a) identify themselves with the purposes of the cluster, (b) exhibit the NACE codes required by the managing organizations, and (c) pay the membership annual/monthly fee.

5 NACE is the abbreviation from *Nomenclature statistique des activités économiques dans la Communauté européenne* and represents the European standard classification of productive economic activities. Particularly, the Iberian Balance Analysis System (SABI database) provides the NACE Rev. 3 classification implemented in 2007. For more information on NACE classification, please refer to: https://www.ine.pt/ine_novidades/semin/cae/CAE_REV_3.pdf.

6 SABI is a secondary database provided by Bureau van Dijk and Informa. It provides data from the Official Commercial Register and additional information such as establishment date, date of status change (active vs. non-active), and international activity. It is a widely used database in research on Portuguese firms (e.g., Mendes et al. 2021b; Silva et al. 2021).

7 Only 8 of the 19 Portuguese clusters were considered for fulfilling the specific requirements for the analysis, introducing some bias in the FBs sample. If it was possible to include all clusters, we would have access to a greater number of family firms formally belonging to these structures, enabling to overcome this issue. However, despite the efforts to obtain all information requested, some managing cluster associations did not participate in the survey due to several reasons. Some were unreachable after multiple attempts, others declined to provide specific data or indicated lack of time to participate.

8 We focused on the timeframe 2010–2018 because SABI database does not provide information for the volume of domestic sales before 2010. This limitation would not allow to calculate the variable “total sales” (i.e., the sum of international and domestic sales) for all the years preceding the reference year (2010). Since our measure of international scale corresponds to the ratio between foreign sales and total sales, requiring the use of domestic sales to estimate the denominator of this ratio, it was not possible to include the years before 2010. Furthermore, although SABI does not directly provides information about foreign sales, the Portuguese version of the database allows to obtain the number of sales and services for both European Union (EU) and non-EU markets. Thus, we extracted the dataset as an Excel file to compute the volume of foreign sales as follows: Foreign sales_t_ = (EU sales_t_ + non-EU sales_t_ + EU services_t_ + non-EU services_t_), where *t* represents the years between 2010 and 2018. In doing so, we used the value “1” for firms revealing foreign sales, at least, in one of the years that comprise our timeframe (2010–2018) and “0” otherwise. The firms coded as “0” were then excluded from the sample.

9 At the date of extraction, the latest year available on SABI was 2018.

10 NUTS stands for *Nomenclature of Territorial Units for Statistics *and represents the level of territorial division for statistical purposes. The Portuguese territory is divided in the following levels: NUTS I consists of 3 groups representing the major socioeconomic regions (*Continente, Região Autónoma dos Açores, Região Autónoma da Madeira*). NUTS II comprises 7 basic regions for the application of regional policies (*Norte, Centro, Área Metropolitana de Lisboa, Alentejo, Algarve, Região Autónoma dos Açores, Região Autónoma da Madeira*). NUTS III is made up of 25 small regions for specific diagnoses (*Unidades Administrativas*). For more information on NUTS classification, please refer to: https://ec.europa.eu/eurostat/web/nuts/background.

11 The post-internationalization speed is a latent variable measured by the observed variables international scale and scope. These are reflective indicators caused by the construct (more precisely, their covariance; Sarstedt et al. 2014), with each indicator reflecting the change in the post-internationalization speed. The post-internationalization speed is, therefore, a reflective construct because it is a trait of indicators (Fornell and Bookstein 1982), their measures are mutually interchangeable (Jarvis et al. 2003) and, if the assessment of the trait change, all indicators will change in a similar manner (Chin 1998).

12 Our study includes a time span from 2010 to 2018. In the timeframe considered, United Kingdom was still an EU member.

13 International scope was coded as follows: 1 = no diversification (only selling to the EU market), 2 = slightly diversified (only selling to non-EU markets), 3 = highly diversified (selling for both markets).

14 This procedure was also followed in previous FBs studies. The criterion of Belenzon and Zarustkie (2012) was used, for example, in the research of Gerulaitiene et al. (2020), McDonald et al. (2017), and Oliveira (2016), while the criterion proposed by Zellweger et al. (2010) was adopted in the studies conducted by Alves and Gama (2020) and Alves (2015).

15 The NACE codes are a nominal qualitative variable used to classify the FBs economic activity and identify the firms that might, eventually, become a cluster member for fulfilling the NACE codes required by the cluster management organizations. Hence, the numerical values related to the NACE codes have no mathematical meaning and, for that reason, cluster affiliation is only measured through the dummy variable (discrete quantitative variable).

16 The *Journal of Family Business Strategy *dedicated in 2014 a special issue recognizing the utility of PLS-SEM in the family business context (Sarstedt et al. 2014). In response to the special issue, FBs researchers have increasingly used PLS-SEM to explore phenomena pertaining to both the business and the family domain (e.g., Pieper et al. 2020; Santiago et al. 2019). These studies exemplify the applicability of PLS-SEM to assess the strength of complex relationships between constructs, as well as the explanatory and predictive power of the models in family firms’ literature (Hair et al. 2021). The combination of PLS-SEM’s capability of providing meaningful results, even on small samples, and the method’s out-of-sample predict power (Chin et al. 2020), makes the method particularly powerful and appropriate for the FBs context (Hair et al. 2021).

17 In the specific case of cluster affiliation, we conclude that only 15.3% of the family firms are cluster affiliated. The same trend is also observable in innovation activities with 16.6% of FBs holding registered brands. This suggests the existence of a highly skewed sample assuming high values with a low frequency (i.e., the number of FBs that do not belong to clusters and do not innovate is higher than those who belong and innovate). Thereby, the lack of distributional assumption was one of the main reasons for choosing PLS-SEM. However, it is worth noting that, in a limited number of situations non-normal data may also influence PLS-SEM results (Sarstedt et al. 2017a). The use of bias-corrected and accelerated (BCa) bootstrapping handles these issues, as it adjusts the confidence intervals for skewness (Efron 1987). Following this guideline, we employed the BCa bootstrapping to correct the data for both bias and skewness (Aguirre-Urreta and Rönkkö 2018).

18 SmartPLS is a software with graphical interface for variance-based structural equation modeling (SEM) using the partial least squares (PLS) to estimate model parameters (Hair et al. 2022). For more information on SmartPLS, please refer to: https://www.smartpls.com.

19 Data groups are generated in SmartPLS by double-checking on the data for the model of interest and selecting “generate data groups”. A name can be specified for the group being generated, and then, the group is established by selecting the dummy variable of interest. After specifying the variable, the data groups are generated (Matthews 2017). The highest-number group (i.e., 1) is used as the reference group. The same applies both in case of single categorical variables and interaction terms.

20 G*Power is a tool to compute statistical power analyses for many different *t* tests, *F* tests,* χ2* tests, and *z* tests. The software can also be used to compute effect sizes and graphically display the results of power analysis. For more information on G*Power, please refer to: https://stats.oarc.ucla.edu/other/gpower/.

21 The *IBM Statistical Package for Social Sciences (SPSS) *software offers advanced statistical analysis, a vast library of machine learning algorithms, text analysis, open-source extensibility, integration with big data and seamless deployment into applications. The SPSS statistics supports a top-down, hypothesis testing approach to the data and, the advanced statistical procedures, help to ensure a high accuracy in research. For more information on SPSS statistics, please refer to: https://www.ibm.com/analytics/spss-statistics-software.

22 Extraction is based on principal components method with an eigen value greater than 1 and maximum iterations for convergence equal to 25 (unrotated factor solution). This method of extraction is adequate when the objective is to summarize most of the original information (variance) in a maximum number of factors, with prediction purposes (Hair et al. 1999).

23 The measurement models determine the relationships between the latent variables (family involvement and post-internationalization speed) and their indicators (family ownership, family management, international scale, international scope). More precisely, each construct has a measurement model (also referred as the outer model in PLS-SEM) that specifies the relationship between each construct and its indicator variables.

24 Model fit indices enable judging how well a hypothesized model structure fits the empirical data. Nevertheless, the notion of model fit known from CB-SEM is not transferable to PLS-SEM as the method follows a different aim when estimating model parameters (the aim is to maximize the explained variance rather than minimize the divergence between covariance matrices) (Hair et al. 2019). Yet, research has brought forward several PLS-SEM based model fit measures, such as the standardized root mean square (SRMR), RMS_theta_, and the exact fit test (Henseler et al. 2014) which, however, have proven ineffective in detecting model misspecifications in settings usually encountered in applied research. Instead of assessing model fit, the structural model assessment in PLS-SEM focuses on evaluating the model’s explanatory and predictive power (Hair et al. 2022).

25 The results of these tests are not shown in the paper but are available from the authors upon request.

26 The implementation of CTA-PLS requires that the measurement models include, at least, four indicators per construct. Thus, we were unable to perform this robustness check since our constructs are measured only by two indicators.

27 FIMIX-PLS segmentation is a method that captures heterogeneity by estimating the probabilities of segment memberships for each observation and, simultaneously, estimates the path coefficients for all segments (Ringle et al. 2015).

28 The FIMIX-PLS segmentation is based on a number of pre-defined segments obtained by estimating the minimum sample size required for the analysis. These segments allow to assess if there are subgroups of data producing substantially different model estimates (Hair et al. 2019).

29 We would like to thank one of the reviewers who suggested the opportunity to obtain further insights by testing the three-way interaction.

30 According to Basco (2013), the demographic approach considers that family involvement (measured by family ownership, family management, family control, family generation) is a sufficient condition to capture family effects on the firm. On the other hand, the essence approach is based on the behavioral perspective and the resource-based view (measured by family resources, brand identity, internal social capital dimensions—i.e., structural, relational, cognitive dimensions) capturing how the family influences firm’s behavior.
