# Supplementary material for: The speed of internationalization in regionally clustered family firms: a deeper understanding of innovation activities and cluster affiliation
Source: Jahrb Reg Wiss. 2023 Mar 14:1–58. Online ahead of print. doi: 10.1007/s10037-023-00182-9 (PMC10013301; doi:10.1007/s10037-023-00182-9)
Supplement: Supplementary file 1 — Robustness Check [file 10037_2023_182_MOESM1_ESM.docx]

**THE SPEED OF INTERNATIONALIZATION IN FAMILY FIRMS: A DEEPER UNDERSTANDING ON INNOVATION ACTIVITIES AND CLUSTER AFFILIATION**

**Robustness Check:**

**Note: Robustness tests information not necessarily to be included in a print version of the article.**

Table 1. Assessment of Nonlinear Effects

| Nonlinear relationship | Coefficient | p-value | Ramsey’s Test |
| --- | --- | --- | --- |
| Family Involvement * Family Involvement 🡪 Post-Internationalization Speed | 0.001 | 0.986 | F (2, 628) = 0.184, p = 0.896 |
| Innovation Activities * Innovation Activities 🡪 Post-Internationalization Speed | -0.056 | 0.457 |  |
| Cluster Affiliation * Cluster Affiliation 🡪 Post-Internationalization Speed | 0.046 | 0.250 |  |
| Interaction Effect 1 * Interaction Effect 1 🡪 Post-Internationalization Speed | 0.028 | 0.304 |  |
| Interaction Effect 2 * Interaction Effect 2 🡪 Post-Internationalization Speed | 0.084 | 0.121 |  |
| Interaction Effect 3 * Interaction Effect 3 🡪 Post-Internationalization Speed | 0.021 | 0.097 |  |
| Firm’s Age * Firm’s Age 🡪 Post-Internationalization Speed | -0.032 | 0.418 |  |
| Firm’s Size * Firm’s Size 🡪 Post-Internationalization Speed | -0.127 | 0.079 |  |
| Performance * Performance 🡪 Post-Internationalization Speed | 0.012 | 0.790 |  |
| Leverage * Leverage 🡪 Post-Internationalization Speed | 0.060 | 0.120 |  |
| Employee R&D Intensity * Employ R&D Intensity 🡪 Post-Internationalization Speed | 0.037 | 0.291 |  |

Interaction effect 1: family involvement * innovation activities; interaction effect 2: family involvement * cluster affiliation; interaction effect 3: family involvement * innovation activities * cluster affiliation.

| **Table 2.** Assessment of Endogeneity  Models | Independent Variables | Coefficient | p-values |
| --- | --- | --- | --- |
| Gaussian copula of model 1 (endogenous variable: family involvement) | Family Involvement*^c^* | 0.006 | 0.873 |
| Gaussian copula of model 2 (endogenous variable: cluster affiliation) | Cluster Affiliation*^c^* | 0.003 | 0.900 |
| Gaussian copula of model 3 (endogenous variable: innovation activities) | Innovation Activities*^c^* | 0.002 | 0.955 |
| Gaussian copula of model 4 (endogenous variable: interaction effect 1) | Interaction Effect 1*^c^* | -0.064 | 0.241 |
| Gaussian copula of model 5 (endogenous variable: interaction effect 2) | Interaction Effect 2*^c^* | -0.023 | 0.718 |
| Gaussian copula of model 6 (endogenous variable: interaction effect 3) | Interaction Effect 3*^c^* | -0.015 | 0.710 |
| Gaussian copula of model 7 (endogenous variable: firm’s age) | Firm’s Age*^c^* | -0.062 | 0.159 |
| Gaussian copula of model 8 (endogenous variable: firm’s size) | Firm’s Size*^c^* | 0.067 | 0.200 |
| Gaussian copula of model 9 (endogenous variable: performance) | Performance*^c^* | 0.016 | 0.711 |
| Gaussian copula of model 10 (endogenous variable: leverage) | Leverage*^c^* | 0.006 | 0.774 |
| Gaussian copula of model 11 (endogenous variable: employee R&D intensity) | Employee R&D Intensity*^c^* | 0.028 | 0.876 |
| Gaussian copula of model 12 (endogenous variables: family involvement, cluster affiliation) | Family Involvement*^c^*  Cluster Affiliation*^c^* | 0.005  0.001 | 0.920  0.984 |
| Gaussian copula of model 13 (endogenous variables: family involvement, innovation activities) | Family Involvement*^c^* | 0.009 | 0.867 |
|  | Innovation Activities*^c^* | -0.004 | 0.940 |
| Gaussian copula of model 14 (endogenous variables: family involvement, interaction effect 1) | Family Involvement*^c^* | 0.040 | 0.375 |
|  | Interaction Effect 1*^c^* | -0.093 | 0.144 |
| Gaussian copula of model 15 (endogenous variables: family involvement, interaction effect 2) | Family Involvement*^c^* | 0.025 | 0.622 |
|  | Interaction Effect 2*^c^* | -0.048 | 0.555 |
| Gaussian copula of model 16 (endogenous variables: family involvement, interaction effect 3) | Family Involvement*^c^* | 0.279 | 0.710 |
|  | Interaction Effect 3*^c^* | -0.041 | 0.811 |
| Gaussian copula of model 17 (endogenous variables: family involvement, firm’s age) | Family Involvement*^c^* | 0.035 | 0.398 |
|  | Firm’s Age*^c^* | -0.072 | 0.139 |
| Gaussian copula of model 18 (endogenous variables: family involvement, firm’s size) | Family Involvement*^c^* | -0.020 | 0.622 |
|  | Firm’s Size*^c^* | 0.062 | 0.242 |
| Gaussian copula of model 19 (endogenous variables: family involvement, performance) | Family Involvement*^c^* | 0.007 | 0.860 |
|  | Performance*^c^* | 0.017 | 0.705 |
| Gaussian copula of model 20 (endogenous variables: family involvement, leverage) | Family Involvement*^c^* | -0.013 | 0.744 |
|  | Leverage*^c^* | 0.235 | 0.621 |
| Gaussian copula of model 21 (endogenous variables: family involvement, employee R&D intensity) | Family Involvement*^c^* | 0.021 | 0.293 |
|  | Employee R&D Intensity*^c^* | 0.039 | 0.832 |
| Gaussian copula of model 22 (endogenous variables: cluster affiliation, innovation activities) | Cluster Affiliation*^c^* | 0.324 | 0.321 |
|  | Innovation Activities*^c^* | -0.485 | 0.324 |
| Gaussian copula of model 23 (endogenous variables: cluster affiliation, interaction effect 1) | Cluster Affiliation*^c^* | 0.044 | 0.172 |
|  | Interaction Effect 1*^c^* | -0.140 | 0.173 |
| Gaussian copula of model 24 (endogenous variables: cluster affiliation, interaction effect 2) | Cluster Affiliation*^c^* | 0.062 | 0.265 |
|  | Interaction Effect 2*^c^* | -0.182 | 0.244 |
| Gaussian copula of model 25 (endogenous variables: cluster affiliation, interaction effect 3) | Cluster Affiliation*^c^* | 0.009 | 0.731 |
|  | Interaction Effect 3*^c^* | -0.051 | 0.769 |
| Gaussian copula of model 26 (endogenous variables: cluster affiliation, firm’s age) | Cluster Affiliation*^c^* | 0.033 | 0.213 |
|  | Firm’s Age*^c^* | -0.086 | 0.124 |
| Gaussian copula of model 27 (endogenous variables: cluster affiliation, firm’s size) | Cluster Affiliation*^c^* | -0.027 | 0.285 |
|  | Firm’s Size*^c^* | 0.049 | 0.373 |
| Gaussian copula of model 28 (endogenous variables: cluster affiliation, performance) | Cluster Affiliation*^c^* | 0.003 | 0.905 |
|  | Performance*^c^* | 0.016 | 0.712 |
| Gaussian copula of model 29 (endogenous variables: cluster affiliation, leverage) | Cluster Affiliation*^c^* | -0.041 | 0.820 |
|  | Leverage*^c^* | 0.052 | 0.766 |
| Gaussian copula of model 30 (endogenous variables: cluster affiliation, employee R&D intensity) | Cluster Affiliation*^c^* | 0.022 | 0.273 |
|  | Employee R&D Intensity*^c^* | 0.052 | 0.766 |
| Gaussian copula of model 31 (endogenous variables: innovation activities, interaction effect 1) | Innovation Activities*^c^* | 0.061 | 0.211 |
|  | Interaction Effect 1*^c^* | 0.119 | 0.165 |
| Gaussian copula of model 32 (endogenous variables: innovation activities, interaction effect 2) | Innovation Activities*^c^* | 0.077 | 0.354 |
|  | Interaction Effect 2*^c^* | -0.153 | 0.320 |
| Gaussian copula of model 33 (endogenous variables: innovation activities, interaction effect 3) | Innovation Activities*^c^* | 0.060 | 0.220 |
|  | Interaction Effect 3*^c^* | -0.012 | 0.752 |
| Gaussian copula of model 34 (endogenous variables: innovation activities, firm’s age) | Innovation Activities*^c^* | 0.046 | 0.249 |
|  | Firm’s Age*^c^* | -0.084 | 0.127 |
| Gaussian copula of model 35 (endogenous variables: innovation activities, firm’s size) | Innovation Activities*^c^* | -0.044 | 0.250 |
|  | Firm’s Size*^c^* | 0.047 | 0.391 |
| Gaussian copula of model 36 (endogenous variables: innovation activities, performance) | Innovation Activities*^c^* | 0.002 | 0.959 |
|  | Performance*^c^* | 0.016 | 0.711 |
| Gaussian copula of model 37 (endogenous variables: innovation activities, leverage) | Innovation Activities*^c^* | 0.006 | 0.821 |
|  | Leverage*^c^* | 0.121 | 0.532 |
| Gaussian copula of model 38 (endogenous variables: innovation activities, employee R&D intensity) | Innovation Activities*^c^* | 0.231 | 0.678 |
|  | Employee R&D Intensity*^c^* | 0.124 | 0.543 |
| Gaussian copula of model 39 (endogenous variables: interaction effect 1, interaction effect 2) | Interaction Effect 1*^c^* | -0.109 | 0.179 |
|  | Interaction Effect 2*^c^* | 0.071 | 0.455 |
| Gaussian copula of model 40 (endogenous variables: interaction effect 1, interaction effect 3) | Interaction Effect 1*^c^* | -0.052 | 0.219 |
|  | Interaction Effect 3*^c^* | 0.154 | 0.421 |
| Gaussian copula of model 41 (endogenous variables: interaction effect 1, firm’s age) | Interaction Effect 1*^c^* | -0.030 | 0.613 |
|  | Firm’s Age*^c^* | -0.055 | 0.118 |
| Gaussian copula of model 42 (endogenous variables: interaction effect 1, firm’s size) | Interaction Effect 1*^c^* | 0.098 | 0.161 |
|  | Firm’s Size*^c^* | 0.045 | 0.397 |
| Gaussian copula of model 43 (endogenous variables: interaction effect 1, performance) | Interaction Effect 1*^c^* | -0.065 | 0.233 |
|  | Performance*^c^* | 0.019 | 0.666 |
| Gaussian copula of model 44 (endogenous variables: interaction effect 1, leverage) | Interaction Effect 1*^c^* | 0.069 | 0.217 |
|  | Leverage*^c^* | -0.108 | 0.723 |
| Gaussian copula of model 45 (endogenous variables: interaction effect 1, employee R&D intensity) | Interaction Effect 1*^c^* | 0.042 | 0.776 |
|  | Employee R&D Intensity*^c^* | -0.032 | 0.154 |
| Gaussian copula of model 46 (endogenous variables: interaction effect 2, interaction effect 3) | Interaction Effect 2*^c^* | 0.152 | 0.403 |
|  | Interaction Effect 3*^c^* | 0.121 | 0.437 |
| Gaussian copula of model 47 (endogenous variables: interaction effect 2, firm’s age) | Interaction Effect 2*^c^* | 0.044 | 0.544 |
|  | Firm’s Age*^c^* | -0.073 | 0.151 |
| Gaussian copula of model 48 (endogenous variables: interaction effect 2, firm’s size) | Interaction Effect 2*^c^* | -0.098 | 0.153 |
|  | Firm’s Size*^c^* | 0.046 | 0.399 |
| Gaussian copula of model 49 (endogenous variables: interaction effect 2, performance) | Interaction Effect 2*^c^* | -0.023 | 0.715 |
|  | Performance*^c^* | 0.016 | 0.707 |
| Gaussian copula of model 50 (endogenous variables: interaction effect 2, leverage) | Interaction Effect 2*^c^* | 0.031 | 0.867 |
|  | Leverage*^c^* | 0.010 | 0.222 |
| Gaussian copula of model 51 (endogenous variables: interaction effect 2, employee R&D intensity) | Interaction Effect 2*^c^* | 0.023 | 0.213 |
|  | Employee R&D Intensity*^c^* | 0.065 | 0.324 |
| Gaussian copula of model 52 (endogenous variables: interaction effect 3, firm’s size) | Interaction Effect 3*^c^* | -0.049 | 0.137 |
|  | Firm’s Size*^c^* | 0.009 | 0.531 |
| Gaussian copula of model 53 (endogenous variables: interaction effect 3, firm’s age) | Interaction Effect 3*^c^* | 0.018 | 0.324 |
|  | Firm’s Age*^c^* | -0.043 | 0.191 |
| Gaussian copula of model 54 (endogenous variables: interaction effect 3, performance) | Interaction Effect 3*^c^* | 0.428 | 0.121 |
|  | Performance*^c^* | 0.050 | 0.213 |
| Gaussian copula of model 55 (endogenous variables: interaction effect 3, leverage) | Interaction Effect 3*^c^* | -0.054 | 0.268 |
|  | Leverage*^c^* | 0.078 | 0.369 |
| Gaussian copula of model 56 (endogenous variables: interaction effect 3, employee R&D intensity) | Interaction Effect 3*^c^* | 0.035 | 0.147 |
|  | Employee R&D Intensity*^c^* | -0.024 | 0.187 |
| Gaussian copula of model 57 (endogenous variables: firm’s age, firm’s size) | Firm’s Age*^c^* | -0.072 | 0.129 |
|  | Firm’s Size*^c^* | 0.057 | 0.273 |
| Gaussian copula of model 58 (endogenous variables: firm’s age, performance) | Firm’s Age*^c^* | -0.062 | 0.160 |
|  | Performance*^c^* | 0.015 | 0.728 |
| Gaussian copula of model 59 (endogenous variables: firm’s age, leverage) | Firm’s Age*^c^* | -0.005 | 0.835 |
|  | Leverage*^c^* | 0.033 | 0.278 |
| Gaussian copula of model 60 (endogenous variables: firm’s age, employee R&D intensity) | Firm’s Age*^c^* | -0.024 | 0.208 |
|  | Employee R&D Intensity*^c^* | 0.013 | 0.666 |
| Gaussian copula of model 61 (endogenous variables: firm’s size, performance) | Firm’s Size*^c^* | 0.067 | 0.198 |
|  | Performance*^c^* | 0.008 | 0.850 |
| Gaussian copula of model 62 (endogenous variables: firm’s size, leverage) | Firm’s Size*^c^* | -0.019 | 0.720 |
|  | Leverage*^c^* | 0.013 | 0.519 |
| Gaussian copula of model 63 (endogenous variables: firm’s size, employee R&D intensity) | Firm’s Size*^c^* | 0.021 | 0.378 |
|  | Employee R&D Intensity*^c^* | 0.037 | 0.771 |
| Gaussian copula of model 64 (endogenous variables: performance, leverage) | Performance*^c^* | 0.056 | 0.213 |
|  | Leverage*^c^* | 0.078 | 0.192 |
| Gaussian copula of model 65 (endogenous variables: performance, employee R&D intensity) | Performance*^c^* | 0.072 | 0.252 |
|  | Employee R&D Intensity*^c^* | -0.057 | 0.333 |
| Gaussian copula of model 67 (endogenous variables: leverage, employee R&D intensity) | Leverage*^c^* | 0.082 | 0.138 |
|  | Employee R&D Intensity*^c^* | 0.132 | 0.456 |
| Gaussian copula of model 68 (endogenous variables: family involvement, cluster affiliation, innovation activities, interaction effect 1, interaction effect 2, interaction effect 3; firm’s age, firm’s size, performance, leverage, employee R&D intensity) | Family Involvement*^c^* | 0.013 | 0.816 |
|  | Cluster Affiliation*^c^* | 0.461 | 0.167 |
|  | Innovation Activities*^c^* | -0.607 | 0.216 |
|  | Interaction Effect 1*^c^* | -0.127 | 0.120 |
|  | Interaction Effect 2*^c^* | -0.079 | 0.629 |
|  | Interaction Effect 3*^c^* | -0.010 | 0.751 |
|  | Firm’s Age*^c^* | -0.072 | 0.161 |
|  | Firm’s Size*^c^* | 0.054 | 0.329 |
|  | Performance*^c^* | 0.010 | 0.811 |
|  | Leverage*^c^* | -0.042 | 0.816 |
|  | Employee R&D Intensity*^c^* | 0.027 | 0.302 |
| *^c^* indicates the Gaussian copulas in the models. Each of the models include all the predictors variables. Interaction effect 1: family involvement * innovation activities; interaction effect 2: family involvement * cluster affiliation; interaction effect 3: family involvement * innovation activities * cluster affiliation. | | | |

Table 3. Assessment of Unobserved Heterogeneity

| Fit Indices | Number of Segments | | | | | | | | |  |
| --- | --- | --- | --- | --- | --- | --- | --- | --- | --- | --- |
|  | 1 | 2 | 3 | 4 | 5 | 6 | 7 | 8 | 9 | |
| AIC | 1758.42 | 1442.61 | 1273.17 | 1256.73 | 1219.63 | 1212.16 | 1150.94 | 1183.04 | 1231.82 | |
| AIC_3_ | 1767.42 | 1461.61 | 1302.17 | 1295.73 | 1268.63 | 1271.16 | 1219.94 | 1262.04 | 1320.82 | |
| AIC_4_ | 1776.42 | 1480.61 | 1288.17 | 1334.73 | 1317.63 | 1330.16 | 1331.94 | 1341.04 | 1409.82 | |
| BIC | 1798.56 | 1527.34 | 1402.51 | 1430.66 | 1438.17 | 1475.16 | 1458.68 | 1535.37 | 1628.82 | |
| CAIC | 1807.56 | 1546.34 | 1431.51 | 1469.66 | 1487.17 | 1534.29 | 1527.68 | 1614.37 | 1717.82 | |
| HQ | 1773.99 | 1475.50 | 1323.38 | 1324.24 | 1304.46 | 1314.30 | 1270.40 | 1319.81 | 1385.82 | |
| MDL_5_ | 2031.11 | 2151.86 | 2018.30 | 2438.41 | 2704.31 | 2999.83 | 3241.61 | 3576.70 | 3928.82 | |
| LnL | -870.21 | -702.30 | -607.59 | -589.36 | -560.82 | -547.08 | -506.47 | -512.52 | -526.91 | |
| EN | n.a. | 0.897 | 0.871 | 0.874 | 0.875 | 0.847 | 0.869 | 0.859 | 0.805 | |
| NFI | n.a. | 0.920 | 0.875 | 0.859 | 0.853 | 0.807 | 0.827 | 0.801 | 0.713 | |
| NEC | n.a. | 65.68 | 82.48 | 80.80 | 79.68 | 97.82 | 83.39 | 89.91 | 124.36 | |
| AIC, Akaike’s information criterion; AIC_3_, modified AIC with factor 3; AIC_4_, modified AIC with factor 4; BIC, Bayesian information criteria; CAIC, consistent AIC; HQ, Hannan Quinn criterion; MDL_5_, minimum description length with factor 5; LnL, Log likelihood; EN, Entropy statistic; NFI, Non-fuzzy index; NEC, Normalized entropy criterion; n.a. = not available; the numbers in bold indicate the best result per segment. | | | | | | | | | |  |
